# Cancer-specific immune evasion and substantial heterogeneity within cancer types provide evidence for personalized immunotherapy

**DOI:** 10.1038/s41698-021-00196-x

**Published:** 2021-06-16

**Authors:** Martin Thelen, Kerstin Wennhold, Jonas Lehmann, Maria Garcia-Marquez, Sebastian Klein, Elena Kochen, Philipp Lohneis, Axel Lechner, Svenja Wagener-Ryczek, Patrick Sven Plum, Oscar Velazquez Camacho, David Pfister, Fabian Dörr, Matthias Heldwein, Khosro Hekmat, Dirk Beutner, Jens Peter Klussmann, Fabinshy Thangarajah, Dominik Ratiu, Wolfram Malter, Sabine Merkelbach-Bruse, Christiane Josephine Bruns, Alexander Quaas, Michael von Bergwelt-Baildon, Hans A. Schlößer

**Affiliations:** 1 Center for Molecular Medicine Cologne, University of Cologne, Faculty of Medicine and University Hospital Cologne, Cologne, Germany; 2 Institute of Pathology, University of Cologne, Faculty of Medicine and University Hospital Cologne, Cologne, Germany; 3 Else Kröner Forschungskolleg Cologne “Clonal Evolution in Cancer”, University of Cologne, Cologne, Germany; 4 Department of Otorhinolaryngology, Head and Neck Surgery, Grosshadern Medical Center, Ludwig Maximilians University, Munich, Germany; 5 Department of General, Visceral, Cancer and Transplantation Surgery, University of Cologne, Faculty of Medicine and University Hospital Cologne, Cologne, Germany; 6 Department of Urology, University of Cologne, Faculty of Medicine and University Hospital Cologne, Cologne, Germany; 7 Department of Cardiothoracic Surgery, University of Cologne, Faculty of Medicine and University Hospital Cologne, Cologne, Germany; 8 Department of Head and Neck Surgery, University of Göttingen, Göttingen, Germany; 9 Department of Head and Neck Surgery, Faculty of Medicine and University Hospital Cologne, Cologne, Germany; 10 Department of Gynecology, University of Cologne, Faculty of Medicine and University Hospital Cologne, Cologne, Germany; 11 German Cancer Consortium (DKTK), Heidelberg, Heidelberg, Germany; 12 Department of Internal Medicine III, University Hospital, Ludwig Maximilians University, Munich, Germany

**Keywords:** Immunoediting, Cancer immunotherapy

## Abstract

The immune response against cancer is orchestrated by various parameters and site-dependent specificities have been poorly investigated. In our analyses of ten different cancer types, we describe elevated infiltration by regulatory T cells as the most common feature, while other lymphocyte subsets and also expression of immune-regulatory molecules on tumor-infiltrating lymphocytes showed site-specific variation. Multiparametric analyses of these data identified similarities of renal and liver or lung with head and neck cancer. Co-expression of immune-inhibitory ligands on tumor cells was most frequent in colorectal, lung and ovarian cancer. Genes related to antigen presentation were frequently dysregulated in liver and lung cancer. Expression of co-inhibitory molecules on tumor-infiltrating T cells accumulated in advanced stages while T-cell abundance was related to enhanced expression of genes related to antigen presentation. Our results promote evaluation of cancer-specific or even personalized immunotherapeutic combinations to overcome primary or secondary resistance as major limitation of immune-checkpoint inhibition.

## Introduction

Immunotherapies targeting immunological checkpoints have become additional pillars for the therapy of hematologic malignancy and solid cancer^1–5^. Release of T-cell inhibition with subsequent enhancement of pre-existing or induction of de-novo anti-tumor immune responses leads to durable clinical benefit in a subset of patients, while the majority does not respond^6,7^. Response prediction is challenging as multiple factors (e.g., antigen presentation, immune checkpoint and neoantigen expression) may enhance or inhibit immune-mediated destruction of tumor cells. Abundance of tumor-infiltrating lymphocytes (TILs), tumor-mutational burden (TMB), gene expression analyses and expression of programmed death ligand 1 (PD-L1) are related to an anti-PD-(L)1 treatment response^8^. Pretherapeutic selection of patients based on TMB or PD-L1 expression was included into FDA drug approvals^9,10^, but these parameters have limited specificity and need further exploration^11–13^. For example, PD-L1 expression on both, host and tumor cells, contributes to PD-(L)1-mediated immune evasion, but PD-L1 expression on TILs is often neglected^14,15^. In view of an increasing number of clinical studies investigating combinations of drugs targeting different immune checkpoints, these observations highlight the translational relevance of immune-modulating molecules in the tumor microenvironment (TME)^16^. While expression of immune-checkpoint ligands on tumor cells has been studied across a wide range of malignancies^17–20^, expression patterns on TILs are only described in very few studies on selected cancers and do not allow comparisons of cancers from different primary sites^21,22^. Available comparative data is mainly derived from transcriptomic analyses and the cellular source of immune-checkpoint targets is often unknown^23^. Moreover, these studies usually do not report on antigen presentation or antigen processing^24,25^.

Here, we report the first cancer- and patient-specific comprehensive analysis of the immune microenvironment and immune evasion including a broader spectrum of cancers. We provide analyses of expression patterns of 34 immune-checkpoint targets in samples obtained from 146 treatment-naïve tumor patients from 10 different types of primary cancer. Our study demonstrates substantial inter-individual variations but also cancer-born patterns.

## Results

### Infiltration by regulatory T cells is the most common feature across cancer types while other lymphocyte subsets show cancer-dependent variation

While changes of lymphocyte subsets have been described for selected cancers, differences between cancer types are poorly understood. We aimed to elucidate cancer-type specific changes of the lymphocyte compartment using flow cytometric analyses of tumor-infiltrating lymphocytes (TILs, *n* = 141), peripheral blood mononuclear cells (CA PBMCs, *n* = 137) and normal tissue (NT, *n* = 89) of previously untreated cancer patients across 10 different cancer types. We additionally included PBMCs of 20 healthy controls (HC PBMCs) to allow comparison of CA PBMCs to their normal counterparts. CD45^+^ lymphocytes in flow cytometry and CD3^+^ cells in immunohistochemistry (IHC) as surrogates of TIL abundance varied substantially between cancer types. Head and neck squamous cell carcinoma (HNSCC) and non-small cell lung cancer (NSCLC) showed the highest infiltration, while it was moderate or low in renal cell carcinoma (RCC), testicular germ cell carcinoma (TGCT), esophago-gastric adenocarcinoma (EGA), colorectal cancer (CRC), hepatocellular carcinoma (HCC), ovarian carcinoma (OVCA), urothelial carcinoma (UCC) and breast carcinoma (BCA) (Supplementary Fig. 1a, b).

**Fig. 1:**
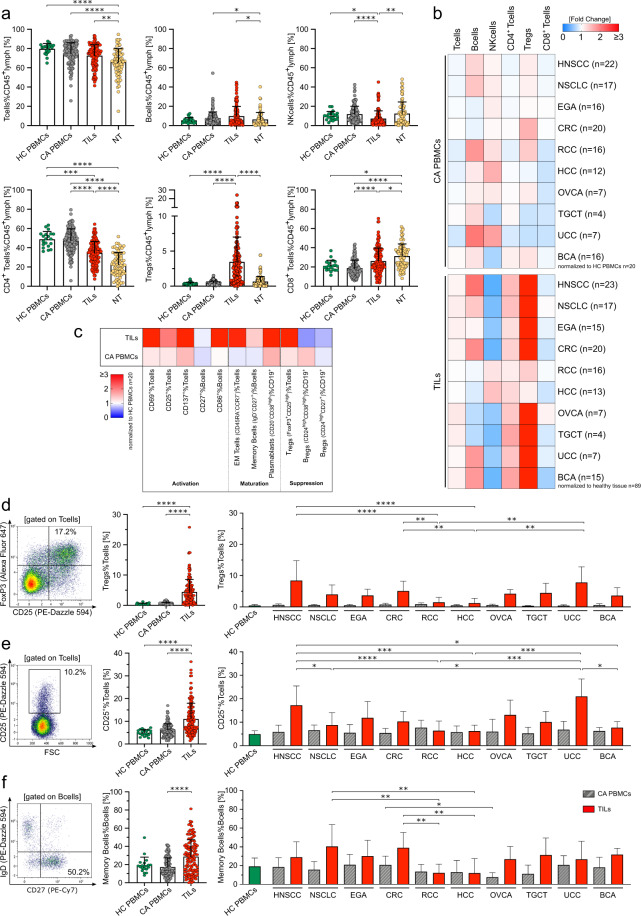
Infiltration by regulatory T cells is the most common feature across different cancer types while other lymphocyte subsets show cancer-dependent variation. **a** Lymphocyte subset in percent of CD45^+^ in healthy control PBMCs (HC PBMCs, *n* = 20), cancer PBMCs (CA PBMCs, *n* = 137), tumor-infiltrating lymphocytes (TILs, *n* = 141) and normal tissue (NT, *n* = 89) were analyzed by flow cytometry. Samples containing <100 CD45^+^ cells were excluded. The gating strategy is described in Supplementary Fig. 12. **b** Cancer-dependent percentages of lymphocyte subsets in CA PBMCs and TILs were normalized to the mean percentages of HC PBMCs or NT, respectively. **c** Relative **c**hange of subsets associated with activation, maturation and suppression in TILs and CA PBMCs normalized to the mean percentage of the respective subset in HC PBMCs. **d**–**f** Representative flow cytometry plots of TILs. Percentages in pooled HC PBMCs, CA PBMCs and TILs and comparison of subsets in TILs and PBMCs between cancer types. **d** CD3^+^CD4^+^CD25^+^FoxP3^+^ regulatory T cells, **e** CD25^+^ activated T cells and **f**, CD27^+^IgD^-^ memory B cells. Significant differences calculated by nonparametric Kruskal–Wallis test followed by Dunn’s post hoc test to correct for multiple comparisons are indicated by asterisks. *p ≤ 0.05, **p ≤ 0.01, ***p ≤ 0.001, ****p ≤ 0.0001. When appropriate, mean ± SD is indicated.

Relative fractions of lymphocyte subsets in PBMCs of cancer patients and healthy controls revealed minor differences (Fig. 1a and b, Supplementary Fig. 2a). In contrast, the immune-cell composition of TILs was distinct to PBMCs and NT. Lymphocyte recovery from normal tissue was low and only allowed analyses of lymphocyte subsets, whereas the majority of tumor samples contained sufficient TILs for detailed analyses. Fractions of T and B cells were increased, while NK cells were decreased in TILs compared to assessable NT (Fig. 1a). Comparison of TILs to CA PBMCs showed an enrichment of CD8^+^ T cells and a decrease of NK cells in the TME (Fig. 1a). CD3^+^CD4^+^CD25^+^FoxP3^+^ Tregs were elevated in TILs compared to HC PBMCs, CA PBMCs and NT (Fig. 1a). Increased Tregs was the most common feature of analyzed TILs and their percentage was significantly higher than in NT for all cancer types despite RCC and HCC (Fig. 1b and Supplementary Fig. 2b). Comparison of lymphocyte differentiation and activation across cancer types revealed cancer-dependent differences for some subsets (e.g., CD25^+^ T cells, CD137^+^ T cells or memory B cells), while others showed less variety (e.g., CD69 or effector memory T cells) (Fig. 1d–f and Supplementary Fig. 3a–e).

### Cancer-specific expression patterns of co-inhibitory and co-stimulatory molecules on TILs

**Fig. 2:**
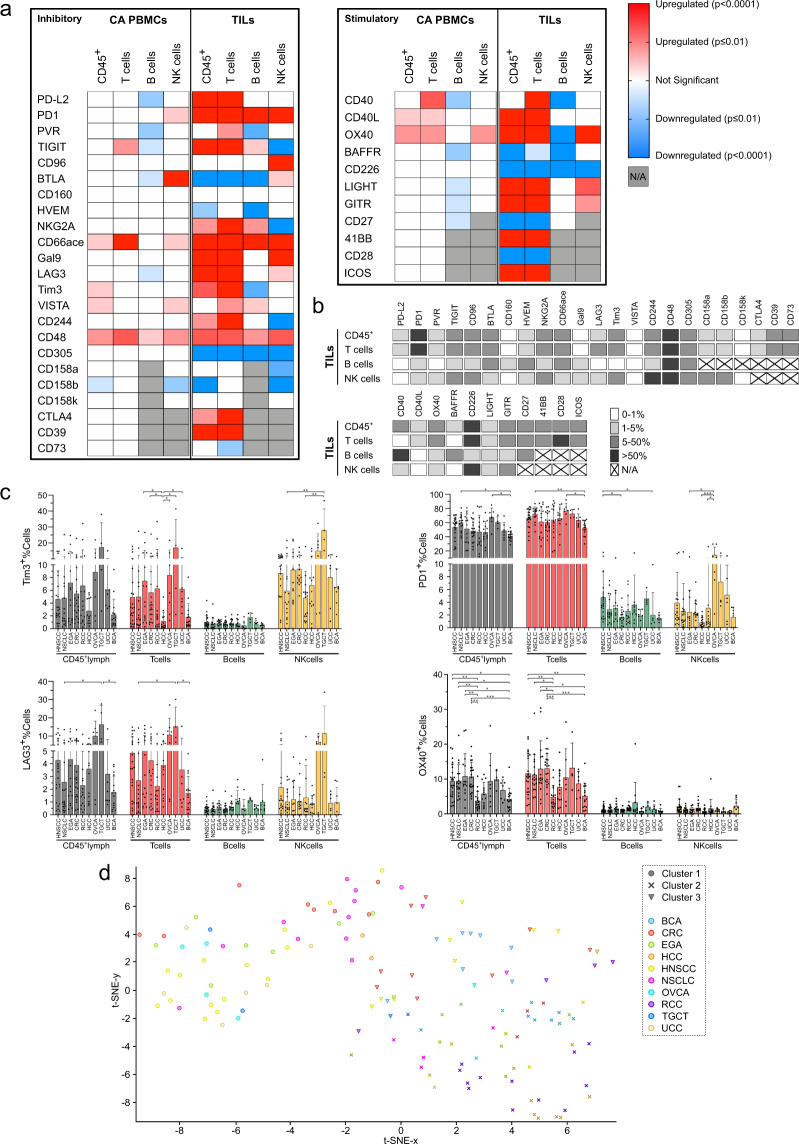
Immune checkpoints on TILs are dysregulated with molecule and cancer-dependent differences. **a** Co-inhibitory and co-stimulatory molecule expression was analyzed by flow cytometry. The gating strategy is described in Supplementary Fig. 12. Significant up-/ downregulation of co-inhibitory and co-stimulatory immune-checkpoint molecule expression on CD45^+^ (*n* = 137), T cells (*n* = 137), B cells (*n* = 120) and NK cells (*n* = 118) of cancer PBMCs (CA PBMCs) and tumor-infiltrating lymphocytes (TILs) compared to the mean expression obtained from healthy control PBMCs (HC PBMCs, *n* = 20). **b** Categorical heatmap showing mean of absolute percentages of immune-checkpoint molecule expression on indicated lymphocyte subsets. **c** Cancer-dependent Tim3, PD1, LAG3, and OX40 expression on the indicated tumor-infiltrating lymphocyte was analyzed by flow cytometry. **d** t-SNE of immune subsets, phenotypes and immune-modulatory molecule expression across cancer types (using NKcells%CD45^+^, Tregs%CD45^+^ and B cells%CD45^+^, CD25^+^%Tcells, Memory%Bcells, CTLA4^+^%Tcells, ICOS^+^%Tcells, GITR^+^%Tcells). Clusters were assigned using Louvain community detection. Significant differences calculated by unpaired two-tailed Mann–Whitney test (**a**) and nonparametric Kruskal–Wallis test followed by Dunn’s post hoc test to correct for multiple comparisons (**c**) are indicated by asterisks. *p ≤ 0.05, **p ≤ 0.01, ***p ≤ 0.001, ****p ≤ 0.0001. When appropriate, mean ± SD is indicated.

Next, we aimed to elucidate cancer-type specific expression patterns of immune-regulatory molecules and their relative abundance on different lymphocyte subsets in peripheral blood and TILs. Expression of 23 co-inhibitory and 11 co-stimulatory molecules was assessed by flow cytometry on bulk CD45^+^ lymphocytes, T, B and NK cells in TILs, CA PBMCs and HC PBMCs. While 32/34 (94%) immune-regulatory molecules were differentially expressed on at least one of the analyzed lymphocyte subsets when comparing TILs to HC PBMCs, only 18/34 (53%) showed differences between CA PBMCs and HC PBMCs. In TILs, 12/23 (52%) co-inhibitory molecules were upregulated on bulk CD45^+^ lymphocytes, 14/23 (61%) on T cells, 6/17 (35%) on B cells and 7/20 (35%) on NK cells. Downregulation of co-stimulatory molecules occurred in 4/11 (36%) on CD45^+^ lymphocytes, 4/11 (36%) on T cells, 4/8 (50%) on B cells and 1/7 (14%) on NK cells (Fig. 2a, for corresponding exact values see Supplementary Table 1). Expression levels were variable with some molecules being expressed by a minor fraction (e.g., CD160, Gal9, VISTA and CD158k) and others by >50% of T, B or NK cells (e.g., PD1, and NKG2A, Fig. 2b). Expression patterns of immune-regulating molecules on TILs showed cancer-specific variations for many molecules (Fig. 2c and Supplementary Figs. 4–6). For example, expression of PD-1 was similar for most cancer types but decreased in BCA. High fractions of LAG3 and Tim3 expressing lymphocytes were common in TGCT and low in BCA and RCC, respectively (Fig. 2c). Mean percentages of positive cells for each molecule on CD45^+^, T, B and NK cells in the different cancer types are shown in supplementary table 2 and illustrated in Supplementary Figs. 4–6. We included immune subsets (Fig. 1a), expression of markers for activation and differentiation (Fig. 1c) and expression of immune-regulatory molecules (Fig. 2a) by using t-distributed stochastic neighbor embedding (t-SNE) analysis to elucidate cancer-dependent patterns of TILs. Following feature selection based on chi-square (χ^2^) selection, the t-SNE analysis revealed 3 clusters with RCC and HCC in one cluster and NSCLC with HNSCC in another cluster. Interestingly, the results were more heterogeneous for other cancer types (Fig. 2d).

### Co-expression patterns of immune-regulatory molecules on immune-cell subsets and cancer cells

**Fig. 3:**
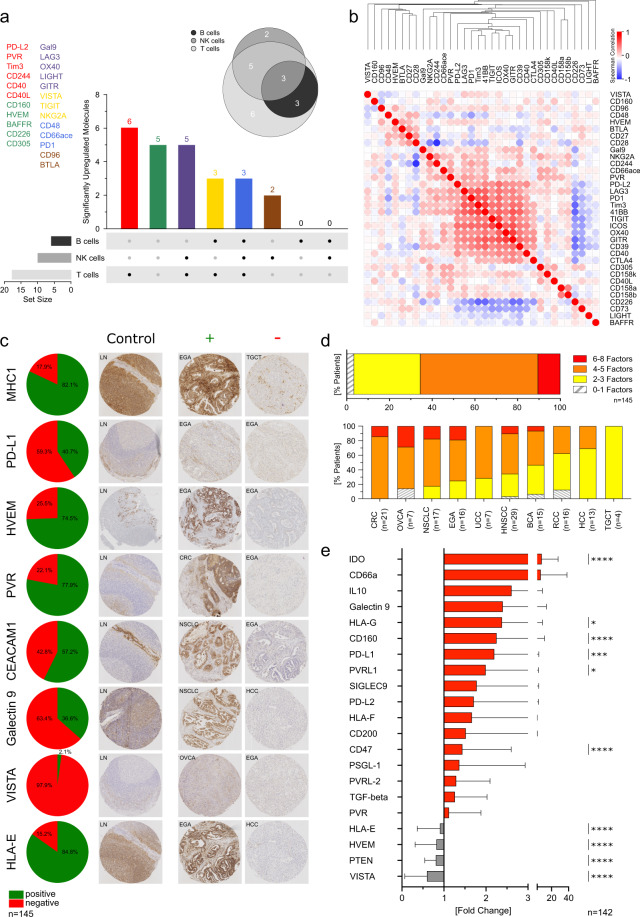
Co-occurrence of multiple escape factors by tumor cells is cancer dependent. **a** Illustration showing the expression pattern and co-occurrence of significantly upregulated molecules on tumor infiltrating B, NK and T cells when compared to healthy control PBMCs. **b** Similarity matrix showing correlations of immune-checkpoint molecule expression on T cells in tumor-infiltrating lymphocytes. Expressions were assessed by flow cytometry. The heatmap was generated using one minus Spearman rank correlation with single linkage. **c** Fractions of patients with MHC class I, PD-L1, HVEM, PVR, CECAM1, Galectin9, VISTA and HLA-E positive or negative tumor cells were determined by tissue micro array (TMA) immunohistochemistry. Representative TMA immunohistochemistry staining controls (healthy lymph node) and positive and negative examples of tumor samples are shown. **d** Percentage of patients expressing multiple immune-escape factors (MHC class I, PD-L1, HVEM, PVR, CECAM1, Galectin9, VISTA, HLA-E) on tumor cells (upper bar chart). Cancer-dependent percentage of patients expressing multiple immune-escape factors on tumor cells (lower bar chart). **e** RNA expression of immune-escape factors within the tumor microenvironment was determined by NanoString. Relative fold change of RNA expression in tumor compared to normal tissue was determined for each patient individually. Mean fold change ± SD is indicated. Significant differences calculated with two-tailed, unpaired Mann–Whitney test (**a**) and two-tailed Wilcoxon matched-pairs signed rank test (**e**) are indicated by asterisks. *p ≤ 0.05, **p ≤ 0.01, ***p ≤ 0.001, ****p ≤ 0.0001.

As ligation of several immune-regulatory molecules may affect responsiveness or activation of more than one lymphocyte lineage, we aimed to assess expression patterns of upregulated immune-modulatory molecules on different TIL subsets^26–28^. Five molecules were not significantly upregulated (CD160, HVEM, BAFFR, CD226, CD305). While differential expression of most molecules was restricted to one or two lymphocyte subsets, PD1, CD66ace and CD48 were upregulated on T, B and NK cells. 6/24 (25%) molecules were increased on T cells and 2/24 (8%) on NK cells only. The remaining molecules were simultaneously upregulated on T and NK cells (5/24, 21%) or T and B cells (3/24, 13%) (Fig. 3a). To identify patterns of co-regulation, we performed hierarchical cluster analyses and spearman correlation of immune-modulatory molecule expression on different lymphocyte subsets. We found a highly correlated upregulation for a set of immune-regulatory molecules on tumor-infiltrating T (Fig. 3b), B and NK cells (Supplementary Fig. 7). For example, expression of Tim3 on T cells appeared highly correlated to that of LAG3, while it was negatively correlated to CD28 (Fig. 3b).

Additionally, expression of ligands for immune-inhibitory molecules on tumor cells in different cancer types was included into our analyses. We found positive staining of tumor cells in IHC staining for VISTA in 2.1% (3/145), Galectin-9 in 36.6% (53/145), PD-L1 in 40.7% (59/145), CEACAM-1 in 57.2% (83/145), HVEM in 74.5% (108/145), PVR in 77.9% (113/145) and HLA-E in 84.8% (123/145) of patients (Fig. 3c)^29–33^. Loss of HLA-I on tumor cells as another important immune-escape mechanisms was detected in 17.9% of included samples (26/145; Fig. 3c). Co-existence of these immune-escape mechanisms was frequently observed (6–8 co-existing factors in 10.3%, 4–5 in 55.2%, 2–3 in 31.0% of patients) with cancer-dependent variety reaching from positivity for combinations of ≤3/8 factors in >60% of RCC, HCC and TCGT to ≥4/8 in >80% of NSCLC, OVC and CRC samples (Fig. 3d, for combinations of co-occurring molecules see Supplementary Fig. 8). In complementary analyses of the tumor microenvironment (TME) on a transcriptional level, we found significant upregulation for 6/21 included immune-regulatory factors in bulk RNA compared to matched normal mucosa. This confirms increased expression of immunosuppressive genes in the TME of included cancers. However, NanoString analyses are not directly comparable to our results of immunohistochemistry or flow cytometry, as they do not allow conclusions regarding the cellular source of increased gene expression (Fig. 3e).

### Impaired expression of genes involved in presentation or processing of antigens is frequent in HCC and NSCLC but rare in RCC

**Fig. 4:**
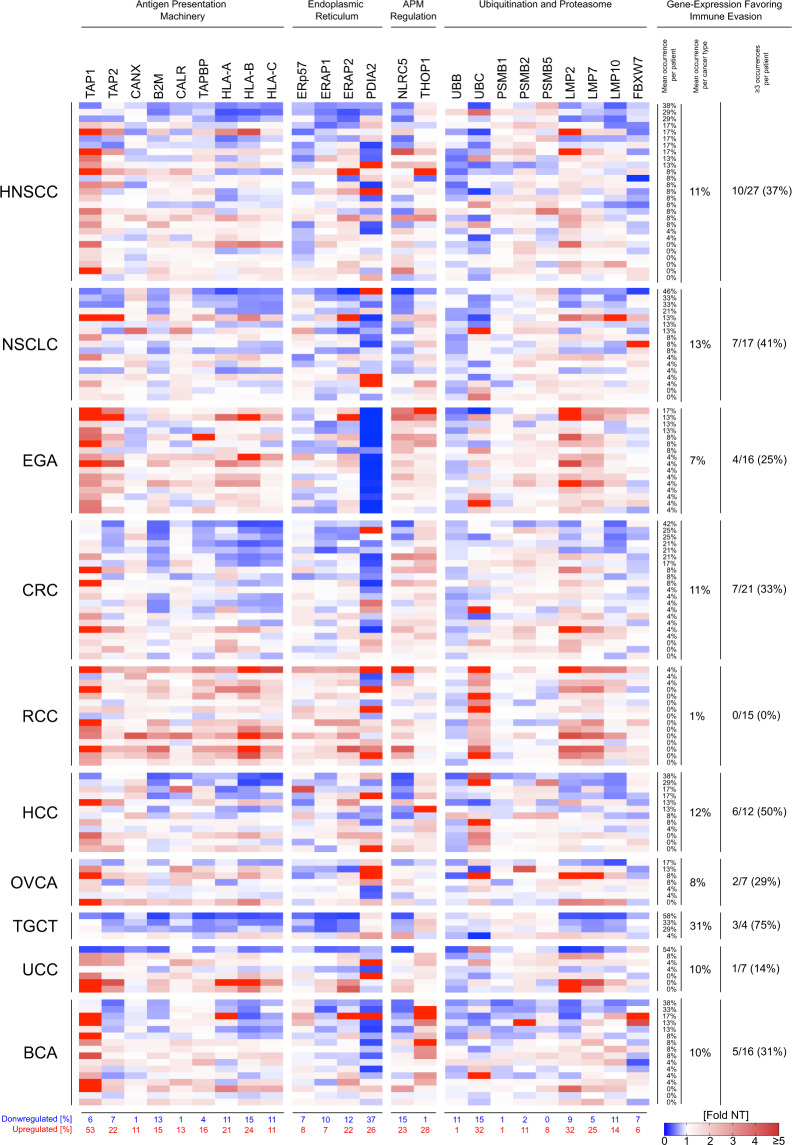
Decreased expression of genes related to antigen presentation is common in most cancer types. NanoString analyses of bulk RNA obtained from formalin fixed paraffin embedded blocks containing tumor or healthy tissue. Fold change represents relative changes in RNA expression for genes associated with antigen presentation in tumor compared to the corresponding normal tissue for each patient individually. A fold change of ≤0.5 was defined as downregulated while a fold change of ≥2 was defined as upregulated. “Mean occurrence per cancer type” was calculated as mean of “Mean occurrence per patient”.

Dysregulation of HLA-I molecules or genes involved in antigen processing is an important immune-escape mechanism in cancer and was also included into our cross-cancer comparison^24,25^. We used NanoString to assess expression of 24 genes involved in antigen-presentation, which were selected based on down-regulation in a fraction of patients for one or more of the included cancer types based on The Cancer Genome Atlas (TCGA) and Genotype-Tissue Expression (GTEx) data using the web server GEPIA (http://gepia.cancer-pku.cn). Despite downregulations in selected patients, mean expression levels for 7/24 genes were increased (>2-fold) in the TME of tumor samples compared to normal tissue. Interestingly, expression of interferon-gamma was related to overexpression of 12/24 genes involved in antigen presentation (Supplementary Fig. 9)^34^. Considering a 2-fold change as cut-off for impaired gene expression^35^, all genes despite *PSMB5* were downregulated in the TME of at least one patient. *B2M*, *HLA-A*, *HLA-B*, *HLA-C*, *ERAP1*, *ERAP2*, *PDIA2*, *NLRC5*, *UBB*, *UBC* and *LMP10* were decreased in >10% of analyzed tumor samples. We found reduced expression of ≥3 (≥12.5%) of included genes in 45/142 patients (32%) and in 33/142 (23%) patients we did not observe alterations. Expression patterns of the 24 genes across different tumor types were heterogeneous. Simultaneous affection of ≥3 (≥12.5%) of included genes was not seen in RCC, while it was found in 75% (TGCT), 50% (HCC), 41% (NSCLC), 37% (HNSCC), 33% (CRC), 31% (BCA), 29% (OVCA), 25% (EGA) and 14% (UCC) of other cancer types (Fig. 4).

### Increased antigen presentation is related to high T-cell infiltrates, while expression of immune-checkpoint molecules is increased in advanced cancers

**Fig. 5:**
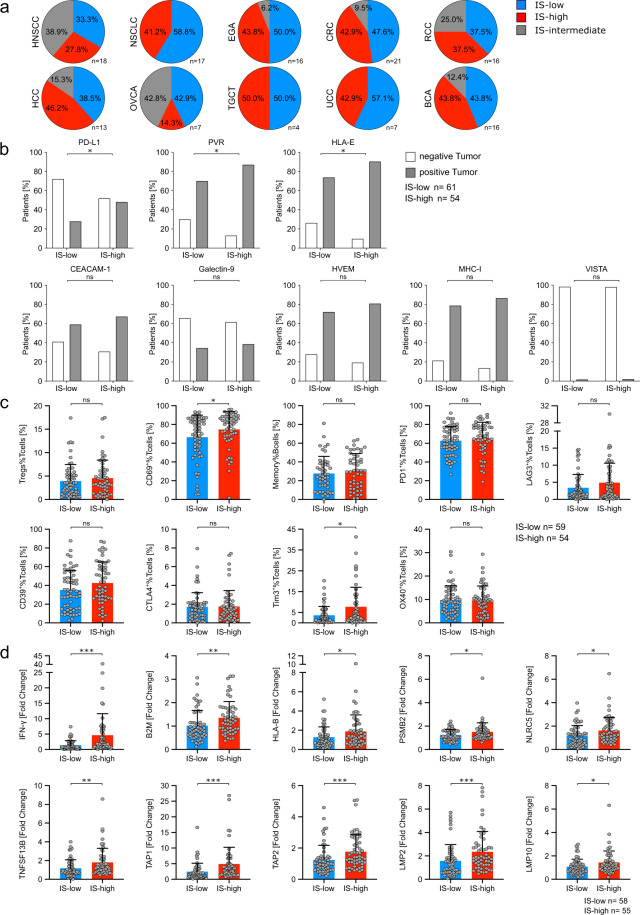
Immune-score high patients show increased antigen presentation. **a** Distribution of immune-score low (IS-low), high (IS-high) and intermediate patients for different cancer types. **b** Tissue-micro array (TMA) immunohistochemistry was performed to determine immune-escape factor occurrence on tumor cells. Percentage of immune-score high/low patients, positive or negative for the respective immune-escape factors are shown. **c** Flow cytometry was performed to determine immune-subset infiltration and immune-regulatory molecule expression on tumor-infiltrating T cells. Percentages of CD4^+^CD25^high^FoxP3^+^regulatory T cells (Tregs%Tcells), CD69^+^ activated T cells (CD69^+^%Tcells), IgD^-^CD27^+^CD19^+^CD20^+^ memory B cells (Memory%Bcells) and the percentage of immune-regulatory molecule expression (PD1, LAG3, CD39, CTLA4, Tim 3 and OX40 in percent of T cells) were stratified according to immune-score low/ high. **d** NanoString RNA expression analysis of tumor and matched normal tissue was performed to determine relative changes for each patient. Bar charts show fold changes in expression in immune-score high/ low patients for the indicated genes. Significant differences calculated with two-sided Fisher’s exact test (**b**), two-tailed unpaired Mann-Whitney test (**c** and **d**) are indicated by asterisks. *p ≤ 0.05, **p ≤ 0.01, ***p ≤ 0.001, ****p ≤ 0.0001. When appropriate, mean ± SD is indicated.

To elucidate differences regarding the composition of immune infiltrates and presence of immune-escape mechanisms in patients with high and low immune infiltrates, we classified tumor samples as immune-score (IS)-high (III–IV), IS intermediate (II) or IS-low (0-I) (Fig. 5a, Supplementary Fig. 10)^36,37^. In IS-high samples, increased expression of PD-L1 (27.9% in IS-low vs. 48.2% in IS-high; p = 0.0336), HLA-E (73.8% vs. 90.7%; *p* = 0.0282) and PVR (70.5% vs. 87.0%; *p* = 0.0415) on tumor cells was detected (Fig. 5b). FACS based immune phenotyping of TILs in IS-high and IS-low patients revealed no differences regarding percentages of lymphocytes subsets and markers for activation or maturation despite CD69 on T cells (Fig. 5c, investigated subsets and molecules in Fig. 1a and c). Expression of most immune-modulatory molecules on T, B and NK cells was similar in IS-high and IS-low samples. Increased percentages of Tim3^+^ T an B cells and CD96^+^ NK cells were the only differences (Fig. 5c, investigated molecules in Fig. 2a). Immune-score stratification of NanoString results revealed increased expression of immune-regulatory cytokines (*IFNG* and *TNFSF13B*) and enhanced expression of 9/24 (37.5%) included genes related to antigen processing and presentation (*B2M*, *TAP1*, *TAP2*, *LMP2*, *LMP10*, *TAPBP* (not shown), *HLA-B*, *PSMB2* and *NLRC5*; Fig. 5d) in IS-high patients.

**Fig. 6:**
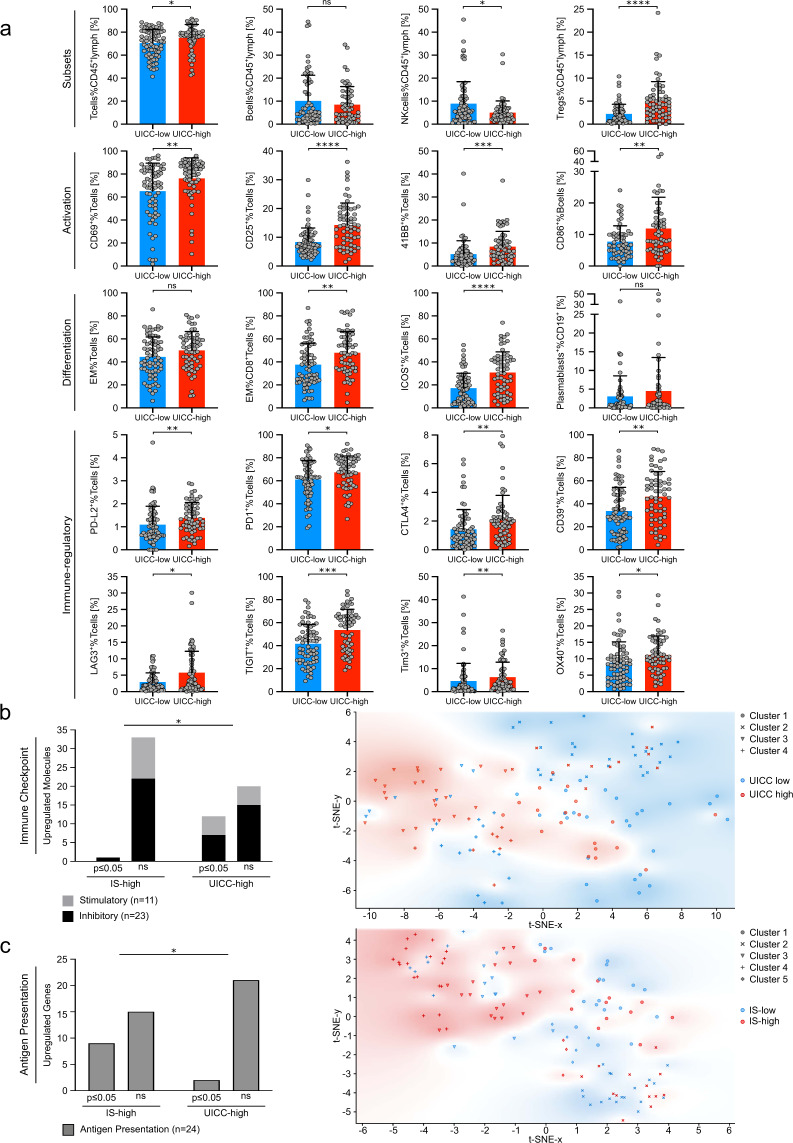
Advanced tumors show accumulated immune evasion. **a** Flow cytometry was performed to investigate immune-subset infiltration, activation, differentiation and the expression of co-inhibitory molecules on tumor-infiltrating T cells. Percentage of the indicated molecules and populations were stratified according to UICC low (UICC I + II) and UICC high (III + IV) patients. **b** Flow cytometry of tumor-infiltrating lymphocytes was performed to investigate immune-checkpoint molecule expression on tumor-infiltrating T cells. The gating strategy is described in Supp. Fig. 12. Number of significantly/ not significantly upregulated co-stimulatory and co-inhibitory molecules in immune-score low vs. high and UICC low vs. high (marker panel in Fig. 2a). t-SNE plot of patients based on their immune-checkpoint molecule expression on tumor-infiltrating T cells (investigated molecules in Fig. 2a). UICC-low patients and UICC-high patients are shown in blue and red, respectively. **c** Number of significantly/ not significantly upregulated genes associated with antigen presentation in immune-score low vs. high and UICC low vs. high (investigated genes in Fig. 4). t-SNE plot of patients based on their expression of genes associated with antigen presentation. Immune-score low and immune-score-high patients are shown in blue and red, respectively. Clusters (**b**, **c**) are assigned using Louvain community detection. Significant differences calculated with two-tailed unpaired Mann–Whitney test (**a**) or two-sided Fisher’s exact test (**b** and **c**) are indicated by asterisks. *p ≤ 0.05, **p ≤ 0.01, ***p ≤ 0.001, ****p ≤ 0.0001. When appropriate, mean ± SD is indicated.

We categorized tumor samples in early (UICC stage I and II, 8^th^ edition) and advanced stages (stage III and IV) to evaluate progression-related changes in the immune TME. Comparison of TILs revealed differences regarding immune-cell subsets, activation, differentiation and expression of immune-modulating molecules. T cells were increased, and NK cells were decreased in advanced stages. Markers for T and B cell activation (CD69, CD25, 41BB and CD86) and T-cell differentiation (CD45RA^-^CCR7^-^ effector memory T cells) were elevated in TILs of advanced stage cancers (Fig. 6a). We also found an increase of Tregs and upregulation of 7/23 (30%, investigated molecules in Fig. 2a) of co-inhibitory molecules on T cells in advanced stages. T cells in the TME of advanced cancer showed increased expression of PD1, CTLA4, CD39, PD-L2, LAG3, TIGIT, Tim3 and decreased expression of CD73 (Fig. 6a, b and Supplementary Fig. 11a). In contrast, NanoString analyses revealed unchanged expression of 21/24 analyzed genes related to antigen presentation (see Supplementary Fig. 11c for differentially expressed molecules). Regarding expression of immune-inhibitory factors, we found expression of HLA-E in 94.2% of advanced stage tumors compared to 76.6% in early stages as only difference (Supplementary Fig. 11b). Strikingly, advanced tumor stages were related to increased expression of immune-modulatory molecules (Fig. 6b), whereas T-cell abundance was related to enhanced antigen presentation (Fig. 6c). This observation was supported by cluster-dependent (Louvain) enrichment of advanced-stage tumor samples in a t-SNE analysis of immune-modulatory molecules and cluster-dependent (Louvain) accumulation of IS-high patients in a t-SNE analyses of genes related to antigen presentation (Fig. 6b and c).

## Discussion

Susceptibility of patients to single agent or combined immunotherapies is highly dependent on the immune microenvironment, which is difficult to assess and poorly defined for most cancers^8,38–40^. Litchfield et al. recently reported about a pooled analysis of transcriptomic and genomic features related to immune-checkpoint therapy response in twelve clinical trials. Their study included data from patients with advanced melanoma, renal cell, lung, urothelial or colorectal cancer and highlighted the predictive role of tumor-cell intrinsic parameters (e.g., tumor mutational burden) as well as parameters related to the immune microenvironment (e.g., CXCL9, CD8A, CXCL13)^41^. This publication further emphasizes the relevance of the immune infiltrate which is usually described in a selected cancer type, whereas studies comparing different types of cancer are rare^42^. One of the few publications covering a broad range of cancers, estimated patterns of TILs based on systematic deconvolution of gene expression data using the TCGA database. In line with our results, this study identified HNSCC, NSCLC and RCC as tumors with the highest lymphocytic infiltrates^23^. Our data provide a comprehensive picture of the immune microenvironment across different cancer types. The composition of lymphocyte subsets in TILs described for our cohort are consistent with available single cancer studies^43,44^. While other lymphocyte subsets showed high variability, an increase of Tregs was a common feature of most included cancer types and the challenging selective targeting of this subset appears broadly applicable^45^.

Expression of ligands for immunosuppressive molecules leads to inactivation of tumor reactive T, B and NK cells and promotes tumor growth^14,18,31,46^. We found cancer-specific variation regarding co-expression of immune-inhibitory ligands by tumor cells. The highest number of co-expressions were detected in CRC and OVCA and the lowest in TCGT, HCC and RCC. The high frequency of naturally occurring co-expressions of immune-inhibitory ligands possibly impairs susceptibility to immunotherapy and may be further increased by immune-checkpoint inhibitors^47,48^. Against the original assumption that expression of PD-L1 on tumor cells is the major immunosuppressive mechanism in the TME, recent studies demonstrate the importance of its expression on non-malignant cells^14,15^. This highly relevant aspect has been neglected for most immune-regulating molecules^49^. One of the few publications addressing expression of immune checkpoints by TILs analyzed six immune checkpoints on T cells in the TME of melanoma. The authors described the highest expression for PD-1, intermediate levels for ICOS, Tim-3 or VISTA and low levels of GITR and OX40^21^. The described percentages are similar to our results for other cancers, and we provide detailed information regarding cellular distribution (T, B and NK cells), co-occurrence and cancer-dependent differences of immune-regulatory molecules. In line with previous results from gene expression data, cluster analyses showed few cancer-dependent patterns and high intra-cancer variability^23,48^. Impaired processing or presentation of antigens has been described for several types of cancer and may also lead to resistance against immunotherapy^24,50,51^. According to our results, impaired expression of antigens is most frequent in the TME of HNSCC, NSCLC, CRC, HCC and TCGT, while it was rare in other cancers. 32% of patients showed downregulation of 3 or more analyzed genes and might be candidates for therapeutic enhancement of antigen presentation^52,53^. Cancer-type dependent patterns and also the clonal distribution of defective antigen processing or presentation merit further investigation (e.g., using single cell RNA sequencing).

Finally, we correlated our analyses of the TME to T-cell abundance and tumor stage as clinically important parameters. Abundance of T cells in the TME is related to prognosis, response to immunotherapy and highly variable within one cancer type^36,49,54^. We found a strong correlation between enhanced expression of genes related to antigen presentation and the immune-score. This aspect needs further investigation as mechanisms underlying “hot” vs. “cold” tumors are poorly understood and comparable data to our unexpected result is scarce^44,55^. Interestingly, the phenotype of immune cells was similar in IS-high and IS-low tumors but frequently different when comparing TILs of patients with early and advanced cancer. This supports an accumulation of multiple immune-escape mechanisms over time as suggested by the cancer immunoediting hypothesis^56^.

Some important limitations of our study have to be addressed in the future. We only included a small number of samples for some tumors and our results need further validation in independent cohorts. We included surgically resected tumor samples, assuming that these relatively large tumor samples better reflect tumor heterogeneity. However, spatial heterogeneity of tumor cells and the immune infiltrate may still be underestimated in our study. We focused our analyses on T, B and NK cells as important lymphocyte effector subsets in cancer. Expression of immune-modulatory molecules by myeloid cells can however be of similar relevance and may also show cancer-type dependent differences in expression patterns, which merit further investigation^57,58^. We selected druggable immune-regulatory molecules and additional pathways may be of similar importance. Single-cell RNA sequencing is a scalable alternative to flow cytometric analyses also allowing conclusions on a single cell level and could provide additional insights into the expression patterns of immune-regulatory molecules in cancer.

The efficacy of anti-PD-L1 plus anti-CTLA-4 highlights the potential benefit of combined immunotherapy and we provide a source for the selection of cancer-dependent or even individualized combinations^12,59,60^. Co-inhibitory gene modules have been described and preclinical studies focusing on synergistic and antagonistic effects of the different targets on immune-cell subsets may identify synergistic combinations of drugs affecting immune modulatory molecules^61^. However, tailored immunotherapeutic combinations based on individualized pretherapeutic evaluation of immune escape as suggested by the “cancer immunogram” may be needed to address the high intratumoral heterogeneity^62,63^.

## Methods

### Patients and samples

146 treatment-naïve cancer patients were included. Samples from peripheral blood (*n* = 137), tumor (*n* = 141), corresponding healthy tissue (*n* = 89) and formalin-fixed paraffin-embedded (FFPE) tumor (*n* = 145) and paired corresponding heathy tissue (*n* = 145) were collected. Patient characteristics are summarized in Supp. Table 3. PBMCs were isolated and tissue specimens were processed to single cell suspensions using a standardized protocol (see Supplementary Methods), resuspended in FBS + 10% DMSO and stored in liquid nitrogen until analysis. Written informed consent was signed by all patients and this study was approved by our institutional ethics committee (no. 17–282).

### Flow cytometry, immunohistochemistry (IHC) and tissue micro arrays (TMAs)

TILs and PBMCs were stained for 10-color flow cytometry (detailed antibody list, Supplementary Table 4). An extensive literature research focusing on druggable targets was performed to select immune-regulatory molecules. Representative literature and clinical studies underlying the classification in co-inhibitory or co-stimulatory molecules is included in Supplementary Table 1. Samples were acquired on a Gallios flow cytometer (Beckman Coulter, USA) and analyzed using Kaluza v2.1 (Kaluza, RRID:SCR_016182, Beckman Coulter, USA; gating strategy in Supplementary Fig. 12). FFPE sections containing tumor tissue and healthy tissue were selected for each patient. The tumor front was delineated digitally on scanned slides using Aperio ImageScope v12.4.0 (Leica, Germany). Whole section slides and tissue micro arrays (∅ 1.2 mm) of tumor specimens were stained on a Leica BOND-MAX or Roche Ventana platform according to the manufacturer’s instructions (details in Supplementary Table 4).

### Automated immune-score analysis

FFPE sections covering the whole cross section of the primary tumor including sufficient adjacent healthy tissue from 135 patients of the cohort were available for IHC analysis of CD3 and CD8 infiltration (details in Supplemnetary Methods).

### RNA isolation and NanoString

For each patient, FFPE sections containing tumor tissue and separate sections of corresponding healthy tissue, furthest from the tumor area, were selected and scratched from slides using a scalpel. RNA was isolated using the Maxwell^®^ RSC RNA FFPE Kit (Promega, USA) and analyzed using NanoString according to the manufacturer’s instructions (details in Supplementary Methods). The selection of genes for the applied customized NanoString was based on relevant targets identified in the systematic literature research. We included ligands for co-inhibitory molecules and soluble immunosuppressive factors, which could not be measured in flow cytometry (e.g., IDO). We additionally selected genes with described functional roles in antigen presentation^64–67^.

### Statistical analyses and visualizations

Non-parametric tests were used as a fraction of samples did not pass the D’Agostino Pearson omnibus-k2 test of normality. Group sizes, levels of statistical significance, definition of error bars and applied tests were included in figure legends. For dimensional reduction using T-distributed Stochastic Neighbor Embedding (t-SNE) we used implementations in Python v3.7 (Python Programming Language, RRID:SCR_008394). Input data for t-SNE was selected after extracting features that differ between groups (feature selection) using a chi-square (x^2^) -test. Input values were normalized, and clustered according to Louvain clustering. Plots were done using Matplotlib (MatPlotLib, RRID:SCR_008624). Detailed information regarding software used for statistical analyses and creation of figures is included in the Supplementary Methods.

### Reporting summary

Further information on research design is available in the Nature Research Reporting Summary linked to this article.

## Supplementary information

Supplementary Data 1

Supplementary Information

Reporting Summary

## Data Availability

The data that support the findings of this study are available from the corresponding author upon reasonable request.
