# Supplementary material for: Cancer-specific immune evasion and substantial heterogeneity within cancer types provide evidence for personalized immunotherapy
Source: NPJ Precis Oncol. 2021 Jun 16;5:52. doi: 10.1038/s41698-021-00196-x (PMC8208982; doi:10.1038/s41698-021-00196-x)
Supplement: Supplementary file 2 — Supplementary Information [file 41698_2021_196_MOESM2_ESM.pdf]

Cancer-specific immune evasion and substantial heterogeneity within cancer types provide evidence for personalized immunotherapy

Martin Thelen[1*], Kerstin Wennhold[1], Jonas Lehmann[1], Maria Garcia-Marquez[1], Sebastian Klein[2,3], Elena Kochen[1], Philipp Lohneis[2], Axel Lechner[4], Svenja Wagener-Ryczek[2], Patrick S. Plum[2,3,5], Oscar Velazquez Camacho[2], David Pfister[6], Fabian Dörr[7], Matthias Heldwein[7], Khosro Hekmat[7], Dirk Beutner[8], Jens P. Klussmann[9], Fabinshy Thangarajah[10], Dominik Ratiu[10], Wolfram Malter[10], Sabine Merkelbach-Bruse[2], Christiane J. Bruns[1,5], Alexander Quaas[2], Michael von Bergwelt-Baildon[1,11,12], and Hans A. Schlößer[1,5]

SUPPLEMENTARY FIGURES

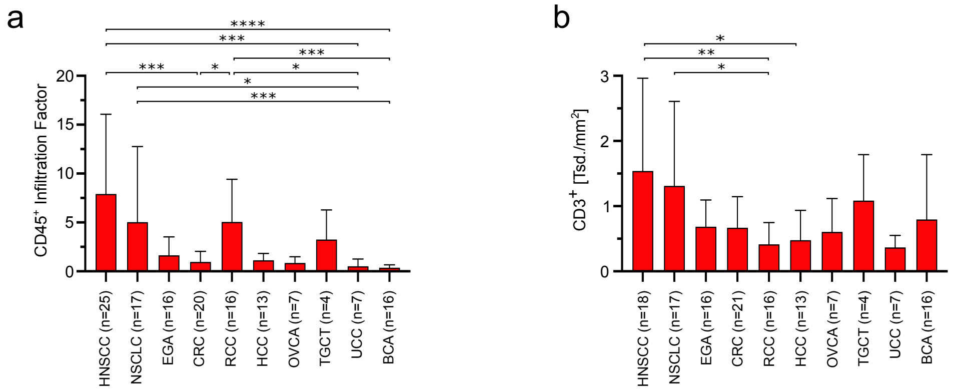

Supplementary Figure 1 Cancer-dependent differences in lymphocyte infiltration of tumor tissue. a Flow cytometry analysis of tumor-infiltrating lymphocytes was performed to determine cancer-dependent lymphocyte infiltration. The gating strategy is described in Supplementary Figure 12. The $CD45^+$ infiltration factor was calculated by dividing the obtained cells in million by the processed tumor tissue in milligram and multiplying with the percentage of $CD45^+$ cells detected by flow cytometry. **b** Digital pathology of whole section CD3 immunohistochemistry was performed to quantify cancer-dependent T-cell infiltration of the tumor per area. Significant cancer-dependent differences calculated by nonparametric Kruskal-Wallis test followed by Dunn's post hoc test to correct for multiple comparisons are indicated by asterisks. * p≤ 0.05, ** p≤0.01, *** p≤0.001, **** p≤0.0001. Mean ± SD is indicated.

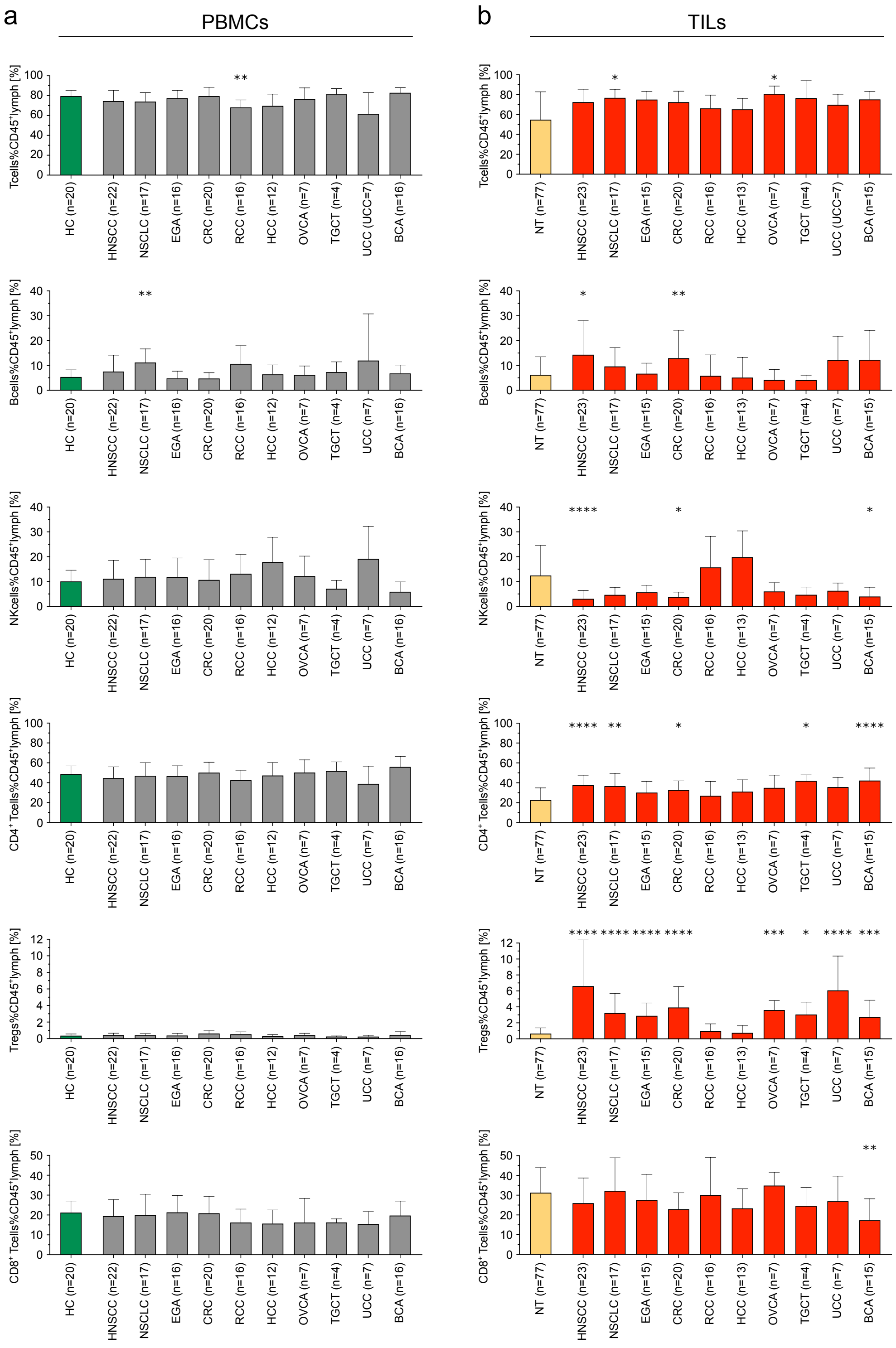

Supplementary Figure 2 Cancer-dependent variation of lymphocyte subsets across different cancer types. a Lymphocyte subsets in percent of CD45⁺ in healthy control PBMCs (HC PBMCs, n=20; green), cancer PBMCs (CA PBMCs, n=137; grey), and **b**, tumor infiltrating lymphocytes (TILs, n=141; red) and normal tissue (NT, n=89; yellow) across different cancer types were analyzed by flow cytometry. Samples containing <100 CD45⁺ cells were excluded. The gating strategy is described in Supplementary Figure 12. Significant differences between HC PBMCs and CA PBMCs (**a**) and NT and TILs (**b**) calculated by nonparametric Kruskal-Wallis test followed by Dunn's post hoc test to correct for multiple comparisons are indicated by asterisks. * p≤ 0.05, ** p≤0.01, *** p≤0.001, **** p≤0.0001. When appropriate, mean ± SD is indicated.

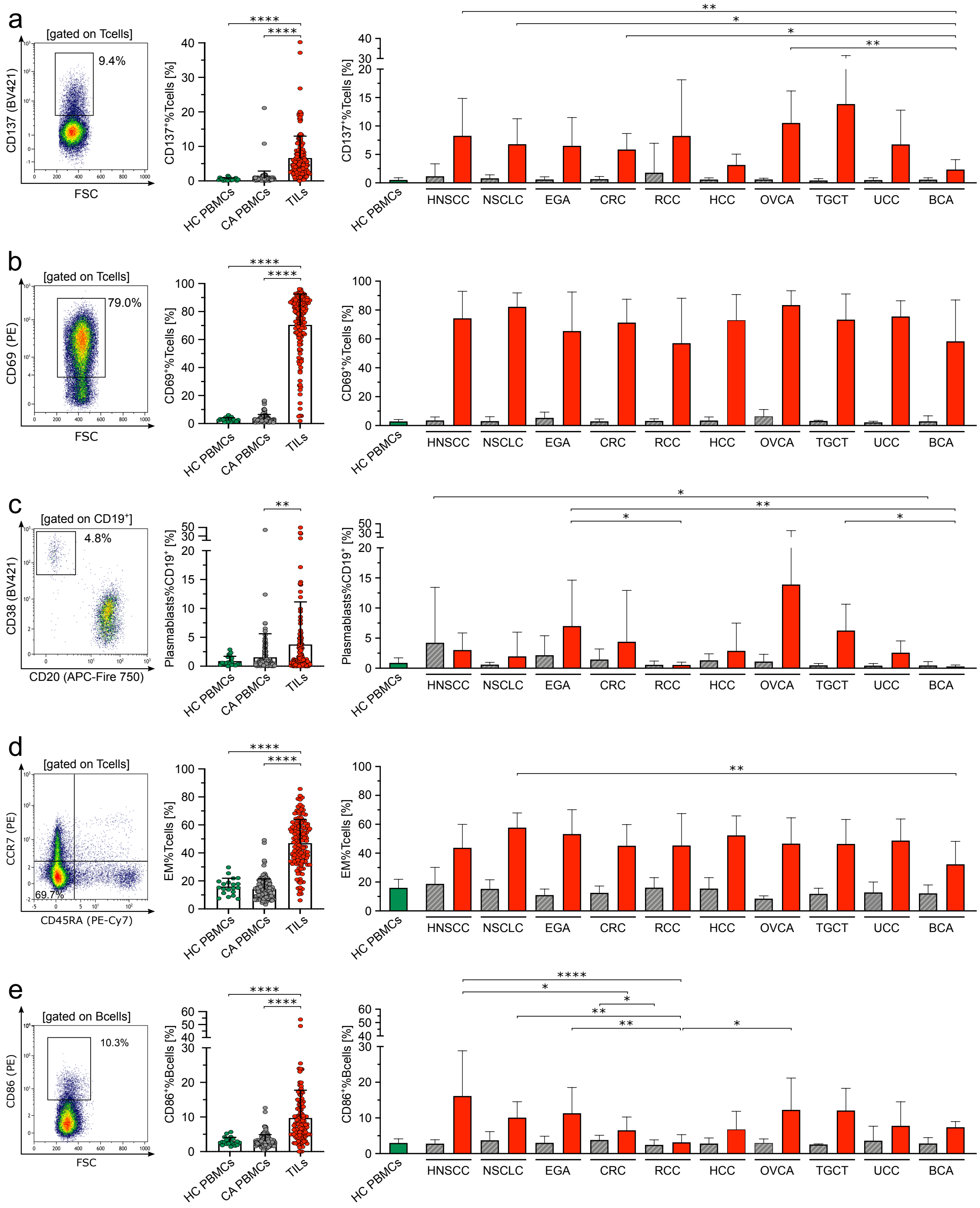

Supplementary Figure 3 Representative flow cytometry plots of TILs with pooled and cancer-dependent infiltration of **a**, CD137$^+$ activated T cells (CD137$^+$%Tcells) **b**, CD69$^+$ activated T cells (CD69$^+$%Tcells) **c**, plasmablasts (CD20$^-$CD38^{high}%CD19$^+$Bcells) **d**, CCR7$^-$CD45RA$^-$ effector memory T cells (CD45RA$^-$CCR7$^-$%Tcells) and **e**, CD86$^+$ activated B cells (CD86$^+$%Bcells). Significant differences in pooled analysis and PBMCs or TILs between different cancer types calculated by nonparametric Kruskal-Wallis test followed by Dunn's post hoc test to correct for multiple comparisons are indicated by asterisks. * p≤ 0.05, ** p≤0.01, *** p≤0.001, **** p≤0.0001. When appropriate, mean ± SD is indicated.

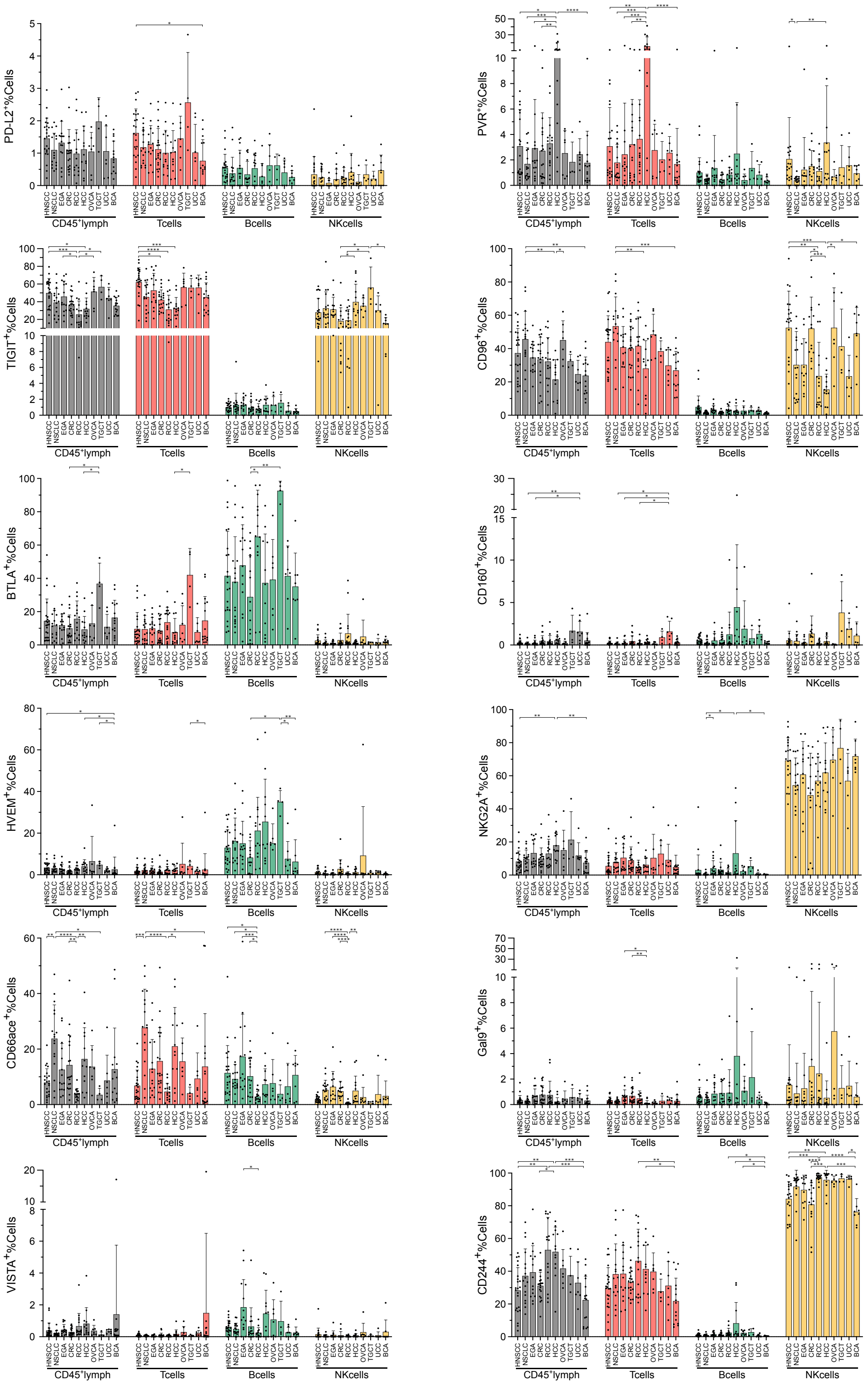

Supplementary Figure 4 Immune-inhibitory checkpoints on tumor-infiltrating lymphocytes (TILs) are dysregulated with marker and cancer-dependent differences. Cancer-dependent PD-L2, PVR, TIGIT, CD96, BTLA, CD160, HVEM, NKG2A, CD66ace, Galectin 9 (Gal9), VISTA, and CD244 expression on the indicated lymphocyte subsets in TILs was analyzed by flow cytometry. The gating strategy is described in Supplementary Figure 12. Significant differences of the indicated lymphocytes subsets between cancer types were calculated by nonparametric Kruskal-Wallis test followed by Dunn's post hoc test to correct for multiple comparisons. Significant differences are indicated by asterisks. *$p \leq 0.05$, **$p \leq 0.01$, ***$p \leq 0.001$, ****$p \leq 0.0001$. When appropriate, mean ± SD is indicated.

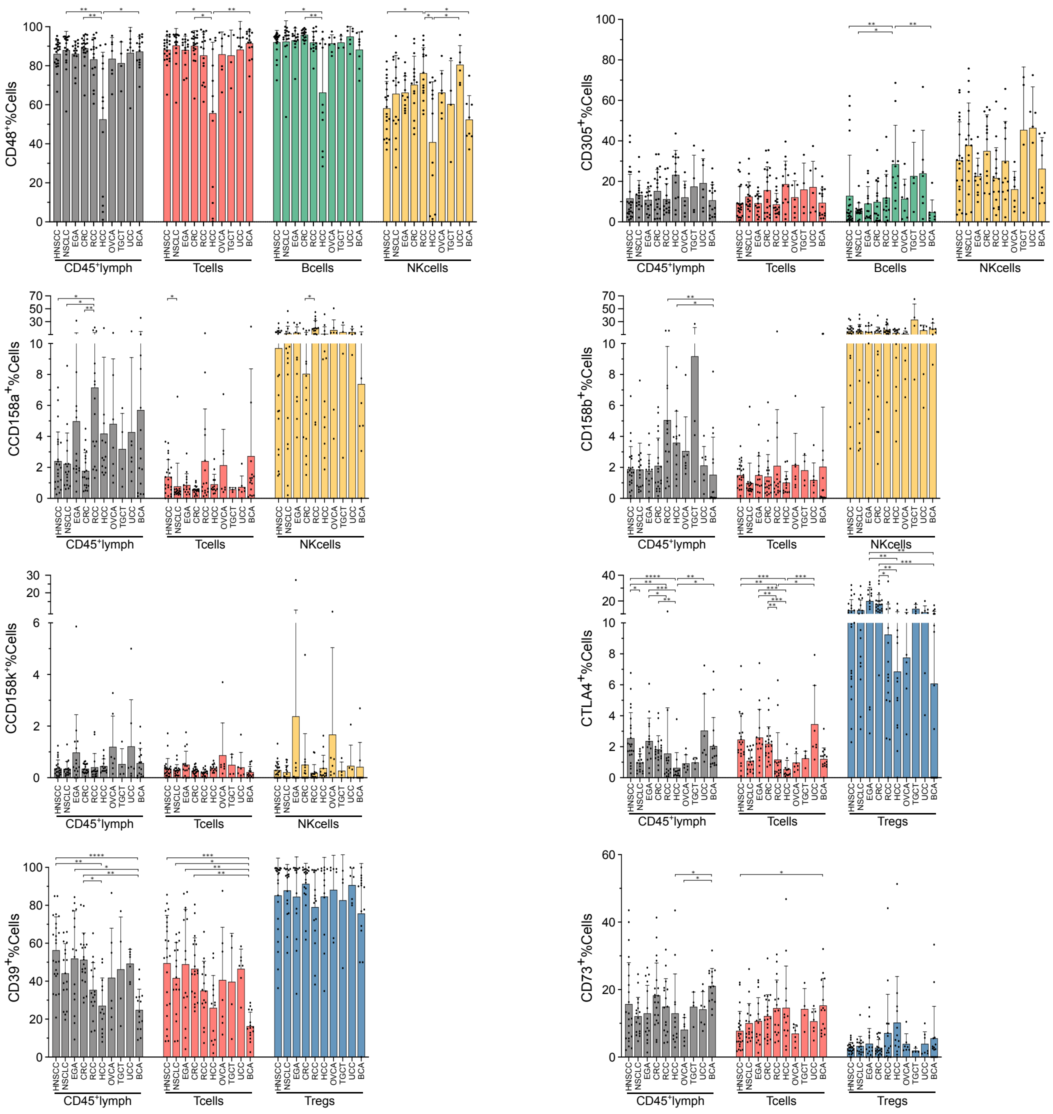

Supplementary Figure 5 Immune-inhibitory checkpoints on tumor-infiltrating lymphocytes (TILs) are dysregulated with marker and cancer-dependent differences. Cancer-dependent CD48, CD305, CD158a, CD158b, CD158k, CTLA4, CD39 and CD73 expression on the indicated lymphocyte subsets in TILs was analyzed by flow cytometry. The gating strategy is described in Supplementary Figure 12. Significant differences of the indicated lymphocytes subsets between cancer types were calculated by nonparametric Kruskal-Wallis test followed by Dunn's post hoc test to correct for multiple comparisons. Significant differences are indicated by asterisks. * p≤ 0.05, ** p≤0.01, *** p≤0.001, **** p≤0.0001. When appropriate, mean ± SD is indicated.

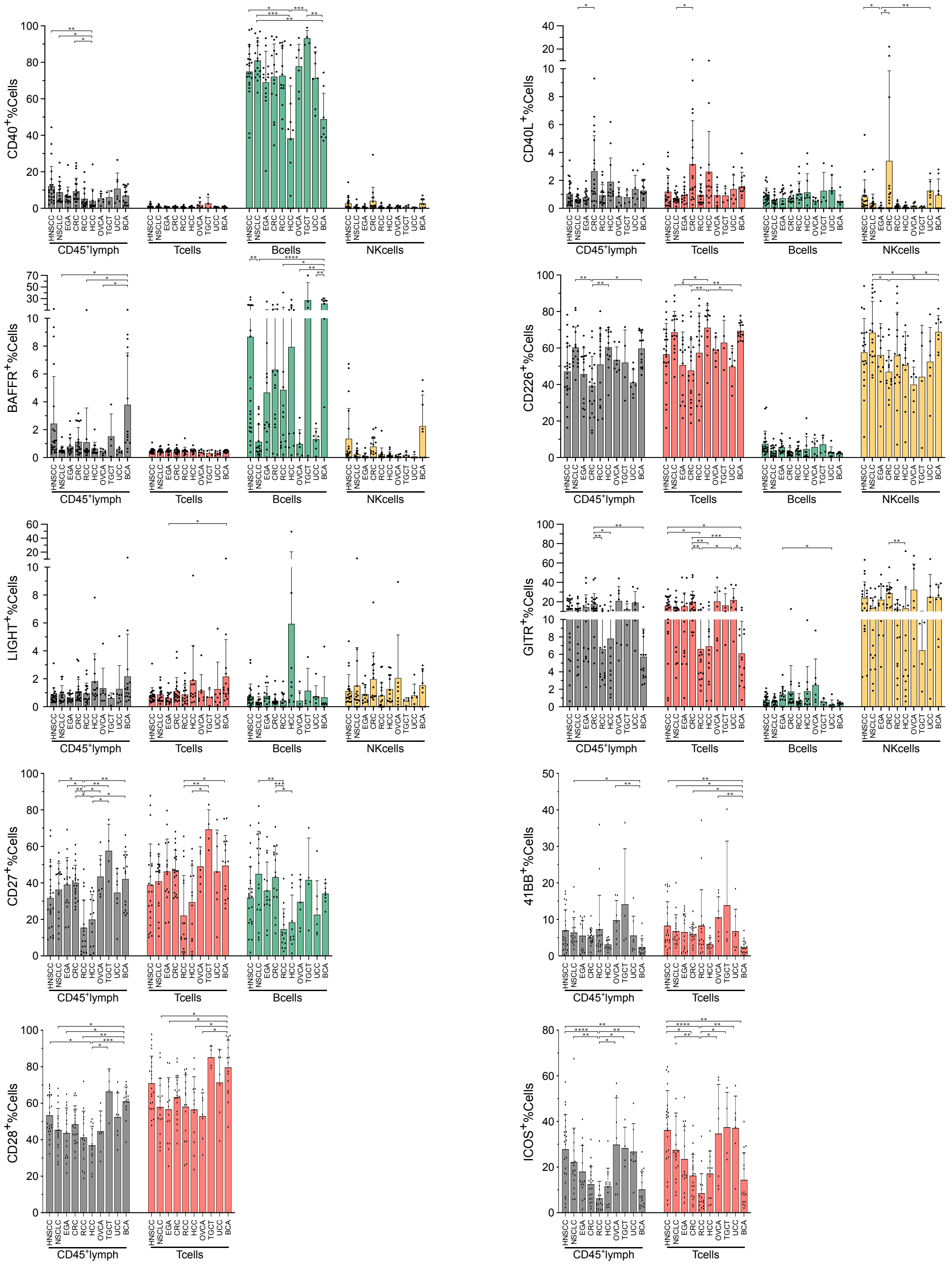

Supplementary Figure 6 Immune-stimulatory checkpoints on tumor-infiltrating lymphocytes TILs are dysregulated with marker and cancer-dependent differences. Cancer-dependent CD40, CD40L, BAFFR, CD226, LIGHT, GITR, CD27, 41BB, CD28, ICOS expression on the indicated lymphocyte subsets in TILs was analyzed by flow cytometry. The gating strategy is described in Supplementary Figure 12. Significant differences of the indicated lymphocyte subsets between cancer types were calculated by nonparametric Kruskal-Wallis test followed by Dunn's post hoc test to correct for multiple comparisons. Significant differences are indicated by asterisks. * p≤ 0.05, ** p≤0.01, *** p≤0.001, **** p≤0.0001. When appropriate, mean ± SD is indicated.

a

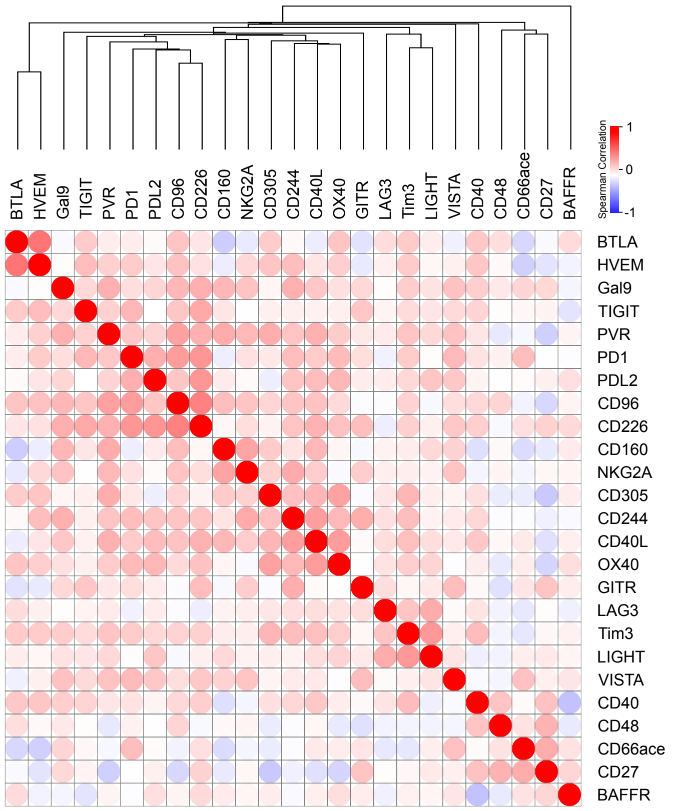

b

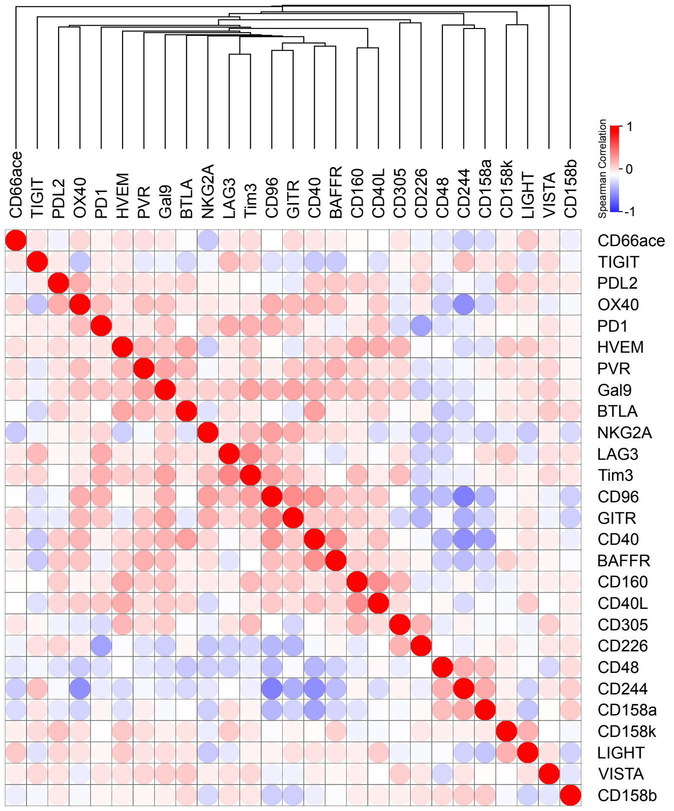

Supplementary Figure 7 Co-occurrence of immune-modulatory molecules on B and NK cells. Immune-modulatory molecule expression on **a**, B cells and **b**, NK cells was hierarchically clustered using single-linkage one minus spearman-rank correlation clustering. Similarity matrix showing spearman-rank correlation.

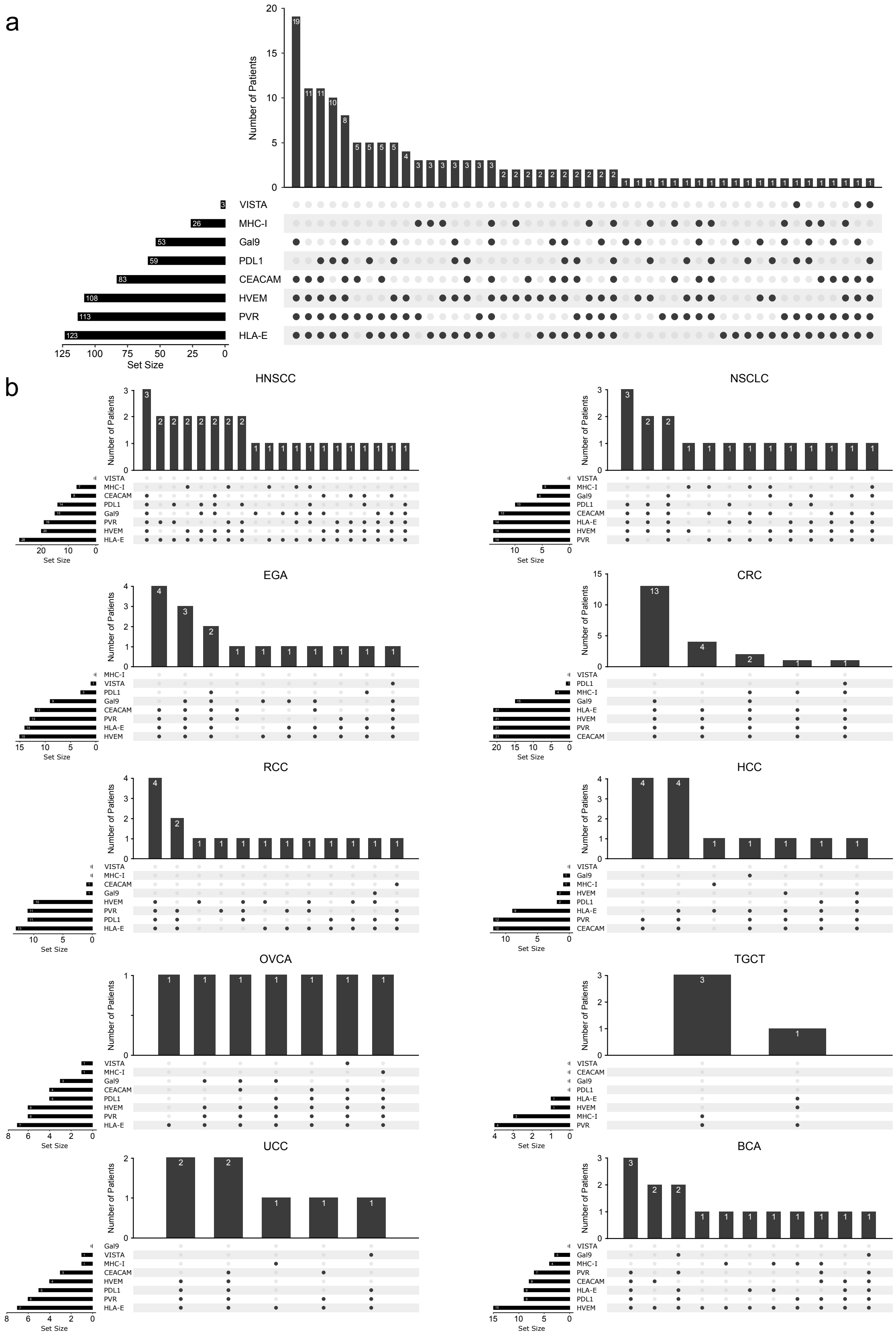

Supplementary Figure 8 Co-occurrence of immune-inhibitory factors on tumor cells. Number of patients with tumor cells, positive for different combinations of the indicated immune-inhibitory factors (MHC- class I loss, PD-L1, HVEM, PVR, CEACAM1, Galectin 9, VISTA, HLA-E) in **a**, pooled analysis (n=145) and **b**, across different cancer types.

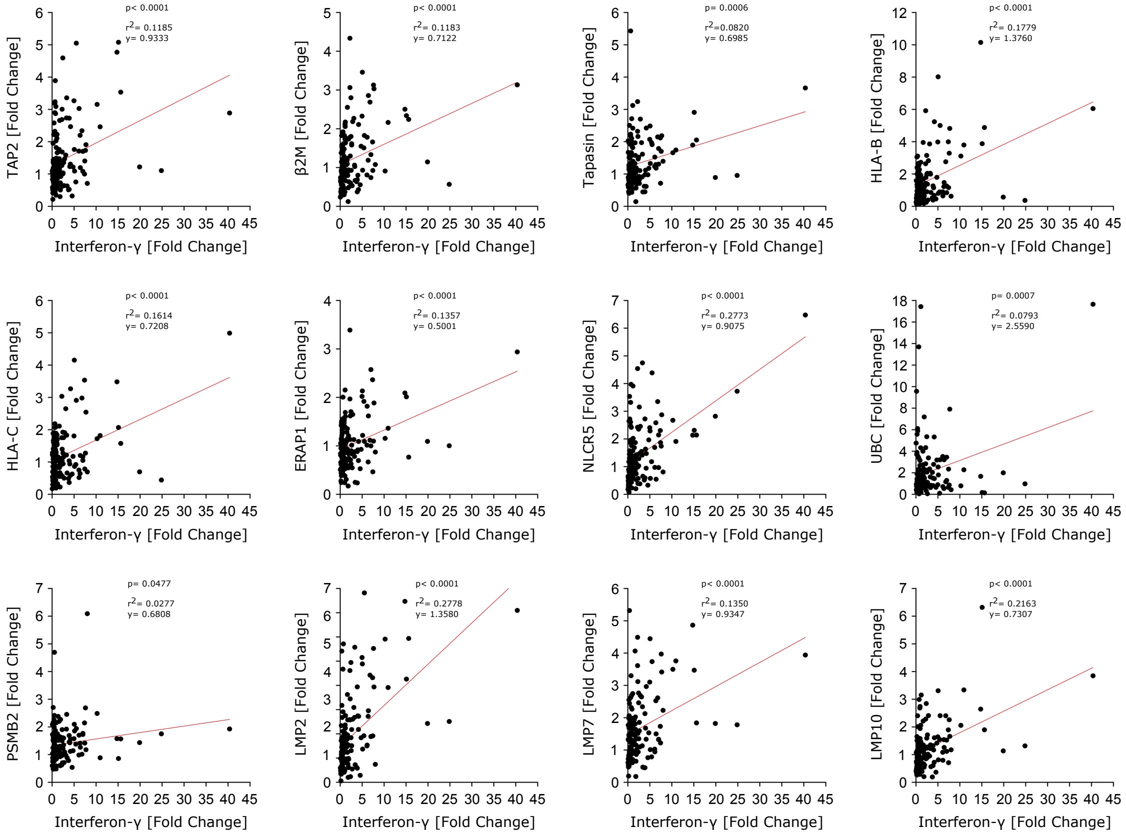

Supplementary Figure 9 Interferon-γ correlates with antigen presentation in the tumor microenvironment. NanoString analyses were performed in bulk RNA of tumor and healthy tissue for each patient (n=142). Fold change represents relative differences of expression in tumor samples and healthy tissue. Plots show correlations of Interferon-γ fold change with genes associated with antigen presentation. Liner regression was calculated, and goodness of fit is indicated by r^2 and y.

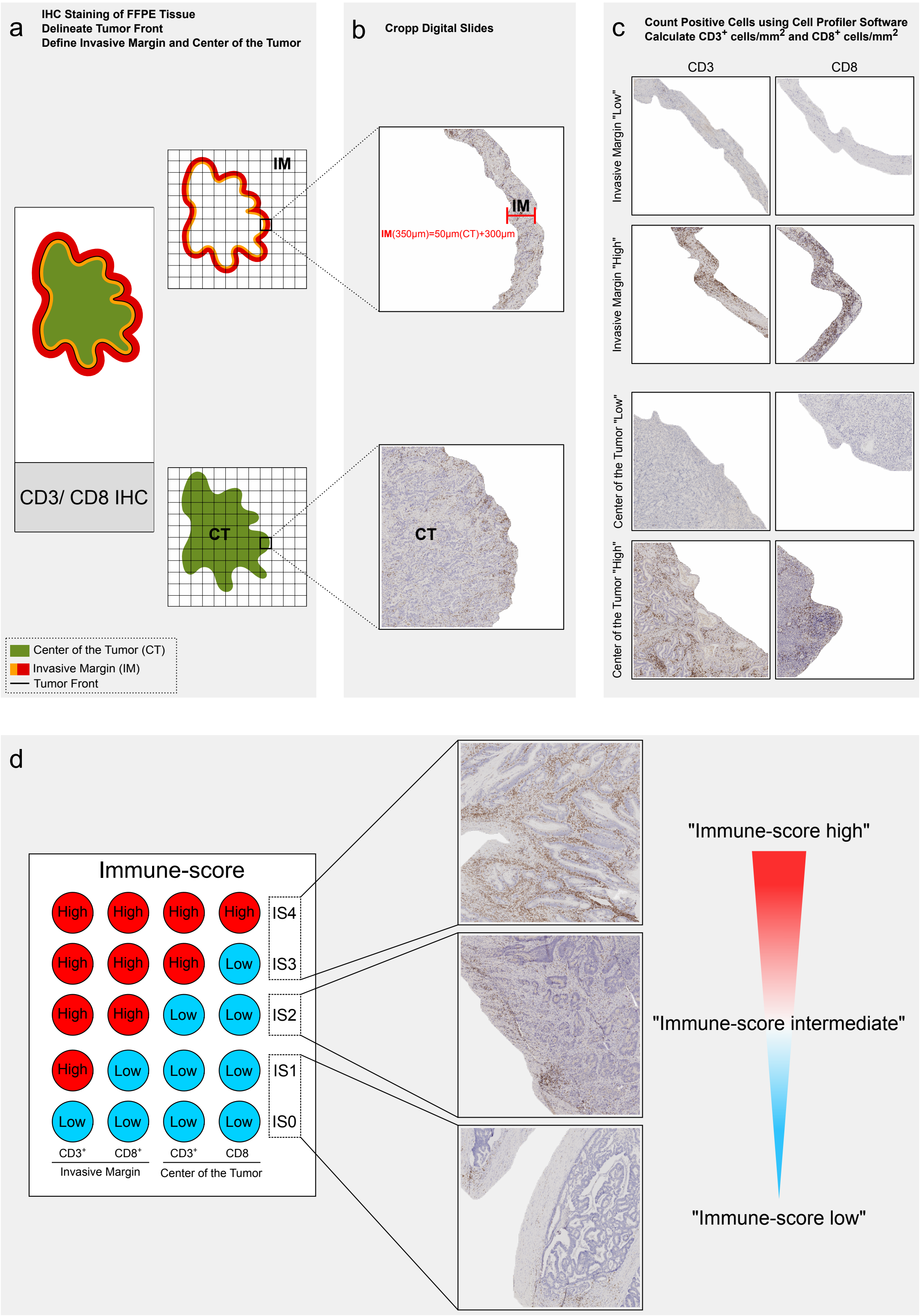

Supplementary Figure 10 Schematic illustrating digital pathology and immune-score generation. **a** Schematics showing the definition of center of the tumor (CT) and invasive margin (IM). **b** Exemplary immunohistochemistry staining of CD3 with CT, IM definition and digital cropping. **c** Exemplary immunohistochemistry staining of CD3 or CD8 showing high and low infiltration in center of the tumor and invasive margin. Infiltration of $CD3^+$ and $CD8^+$ cells per area (cells/mm^2) were calculated using CellProfiler software. **d** Table showing immune-score categories. Representative tiles of immune-score high (IS3 and IS4), immune-score intermediate (IS2) and immune-score low (IS0 and IS1).

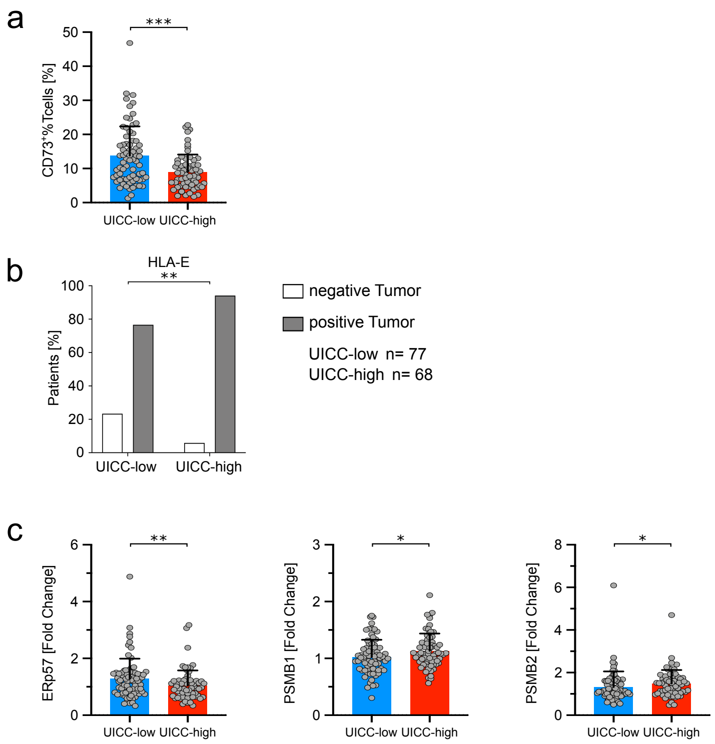

Supplementary Figure 11 Effect of tumors stage on the expression of immune-inhibitory factors and antigen presentation in the tumor microenvironment. a Immune-modulatory molecule expression on tumors-infiltrating lymphocytes was analyzed using flow cytometry. The gating strategy is described in Supplementary Figure 12. Percentage of $CD73^{+}$ tumors-infiltrating T cells in UICC high (UICC state 3+4) versus UICC low (UICC state 1+2) is shown. **b** Immunohistochemistry was performed on tissue micro arrays to determine expression of immune-inhibitory factor by tumors cells. Percentage of UICC high/low patients with HLA-E positive or negative tumors is shown. **c** NanoString RNA expression analysis of tumors and matched normal tissue was performed to determine relative changes for each patient. Bar charts show fold changes in expression stratified by immune-score high/ low for the indicated genes. Significant differences calculated with two-tailed Mann-Whitney test (**a** and **c**) or two-sided Fisher's exact test (**b**) are indicated by asterisks. * p≤ 0.05, ** p≤0.01, *** p≤0.001, **** p≤0.0001. When appropriate, mean ± SD is indicated.

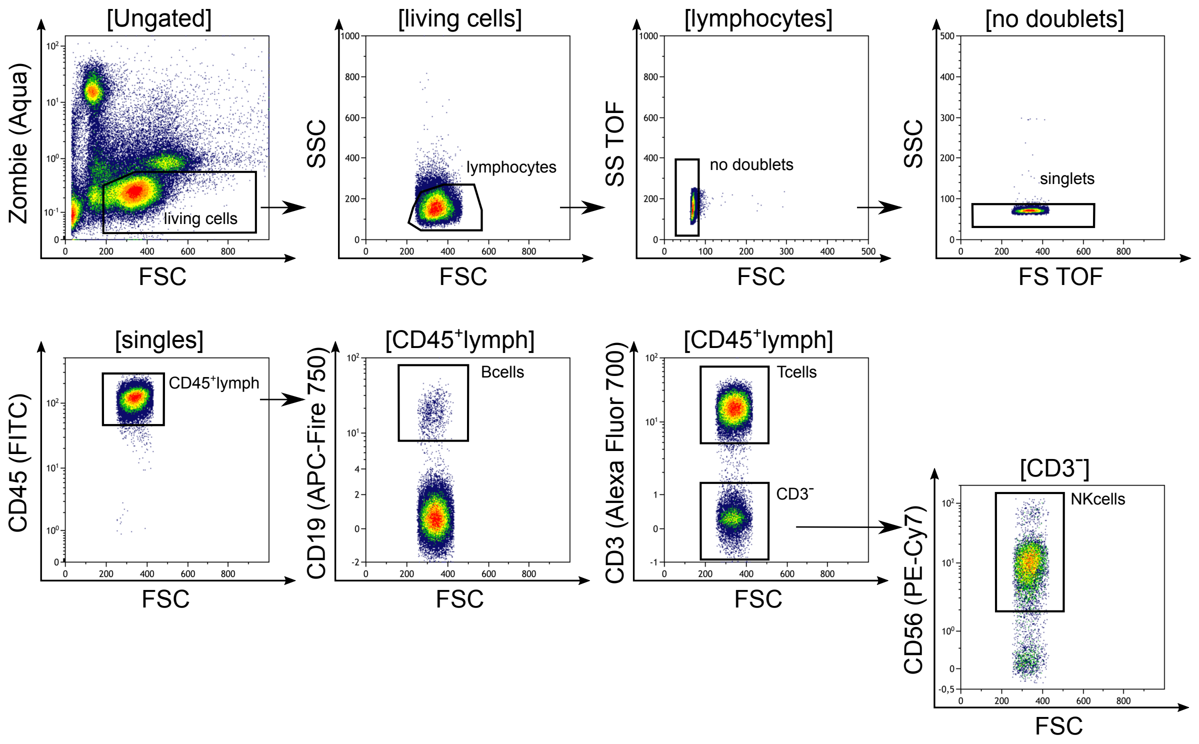

Supplementary Figure 12 Detailed flow cytometry gating strategy of immune subset linages. Dead cells were excluded (living cells). Lymphocytes were selected by gating for size and granularity of the event (Forward Scatter (FCS) versus Side Scatter (SSC), respectively). Living lymphocytes were further plotted by Side Scatter Time-of-Flight (SS TOF) versus FSC and SSC versus Forward Scatter Time-of-Flight (FS TOF) to exclude doublets and gate single cells. From the single cell gate (singlets), $CD45^+$ lymphocytes were gated by CD45 expression ($CD45^+$lymph). B cells were defined by gating on $CD45^+$lymph cells and the expression of CD19. T cells were defined by gating on $CD45^+$lymph cells and the expression of CD3 whereas $CD3^-$ cells expressing CD56 were considered as NK cells. Detailed list of antibodies, clones and fluorochromes is shown in Supplementary Table 4.

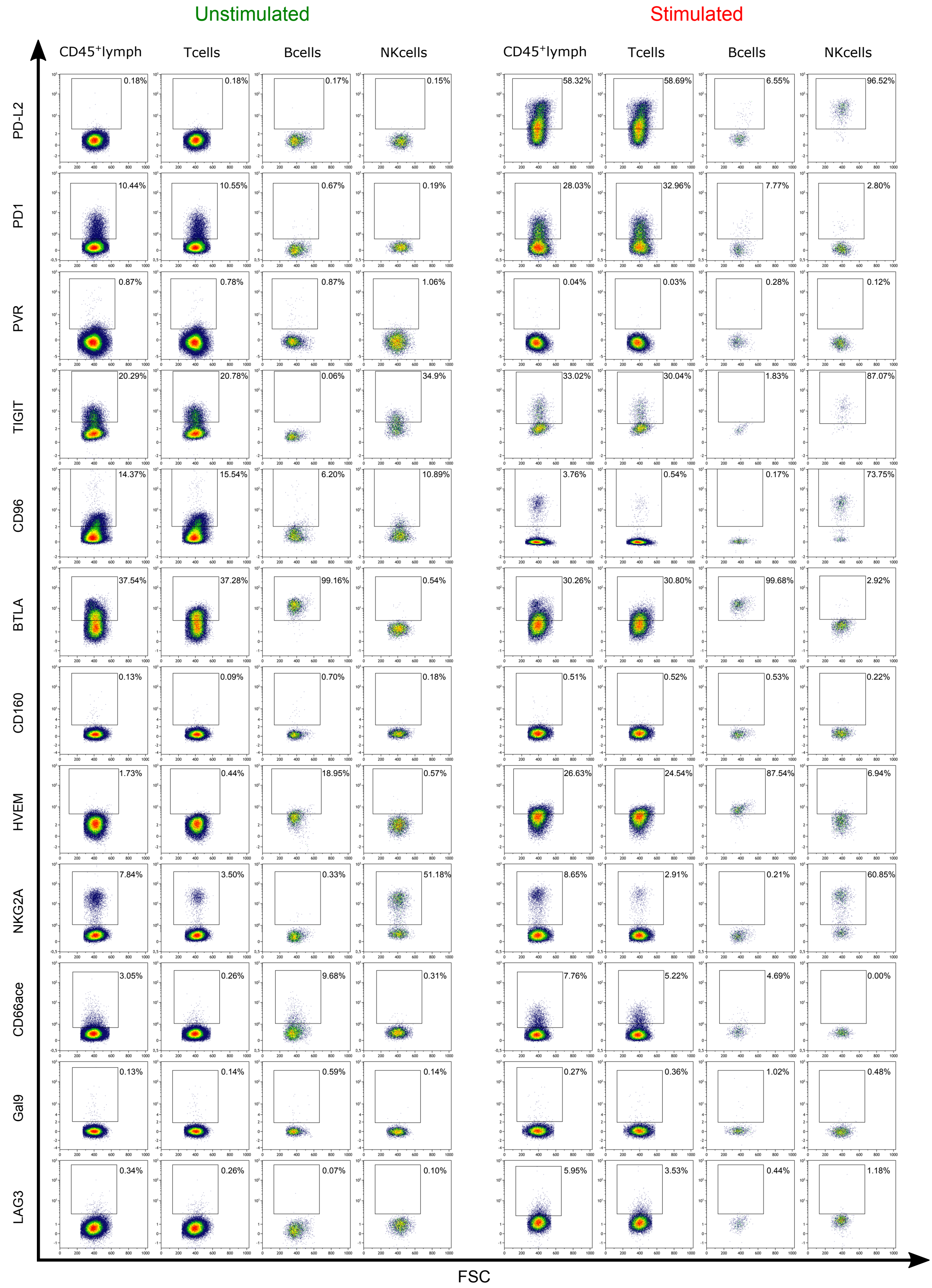

Supplementary Figure 13 Representative flow cytometry plots of co-inhibitory molecule expression (PD-L2, PD-1, PVR, TIGIT, CD96, BTLA, CD160, HVEM, NKG2A, CD66a, Gal9, LAG3) on unstimulated and stimulated healthy control PBMCs. Flow cytometry analyses of co-inhibitory molecule expression on the indicated immune subsets was performed before and after stimulation with anti-CD3 (Biolegend, USA, clone OKT3, 0.5 µg/ml) and anti-CD28 (Biolegend, USA, clone CD28.2, 2 µg/ml) for two days. The gating strategy is described in Supplementary Figure 12.

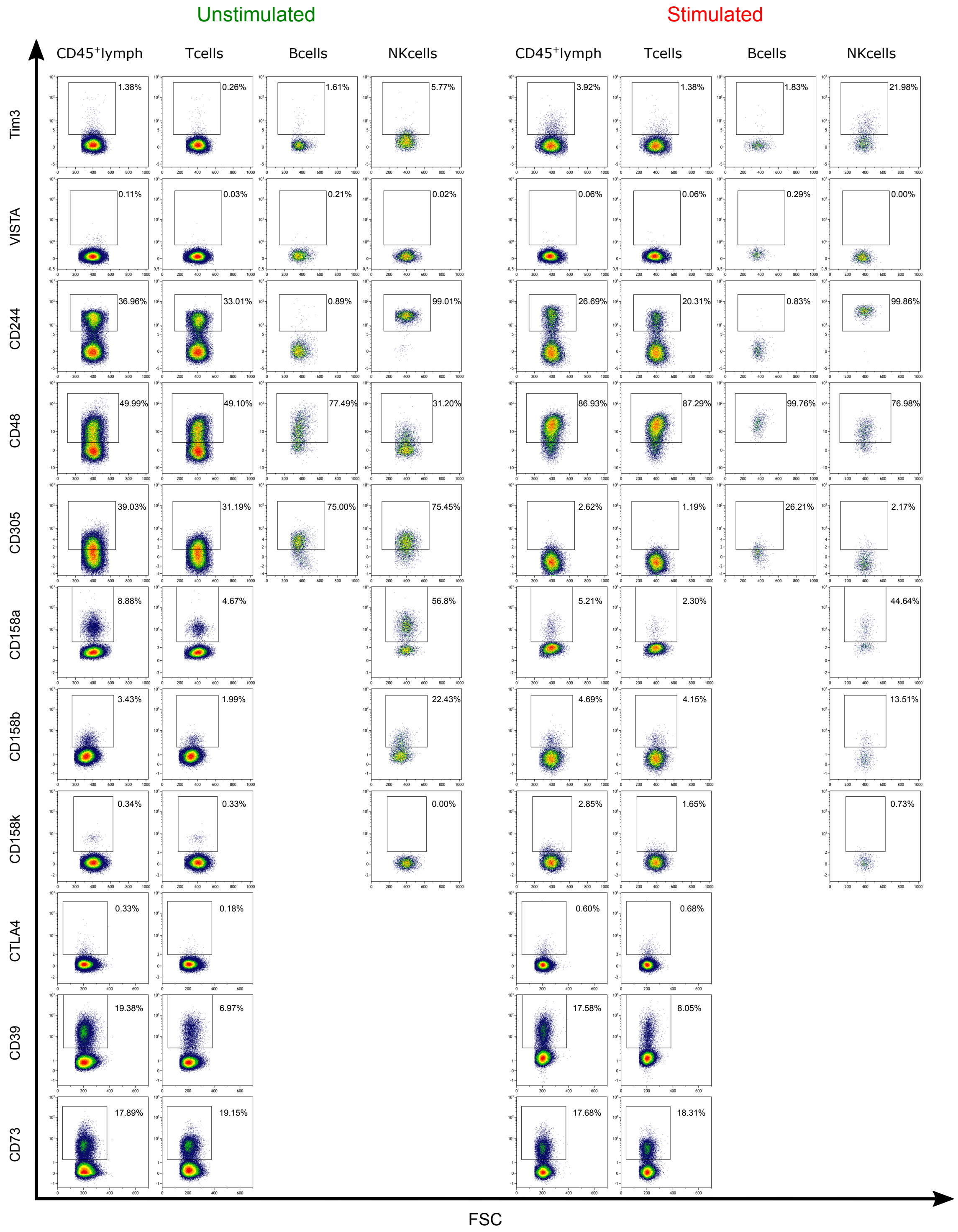

Supplementary Figure 14 Representative flow cytometry plots of co-inhibitory molecule expression (Tim3, VISTA, CD244, CD48, CD305, CD158a, CD158b, CD158k, CTLA4, CD39, CD73) on unstimulated and stimulated healthy control PBMCs. Flow cytometry analyses of co-inhibitory molecule expression on the indicated immune subsets was performed before and after stimulation with anti-CD3 (Biolegend, USA, clone OKT3, 0.5 µg/ml) and anti-CD28 (Biolegend, USA, clone CD28.2, 2 µg/ml) for two days. The gating strategy is described in Supplementary Figure 12.

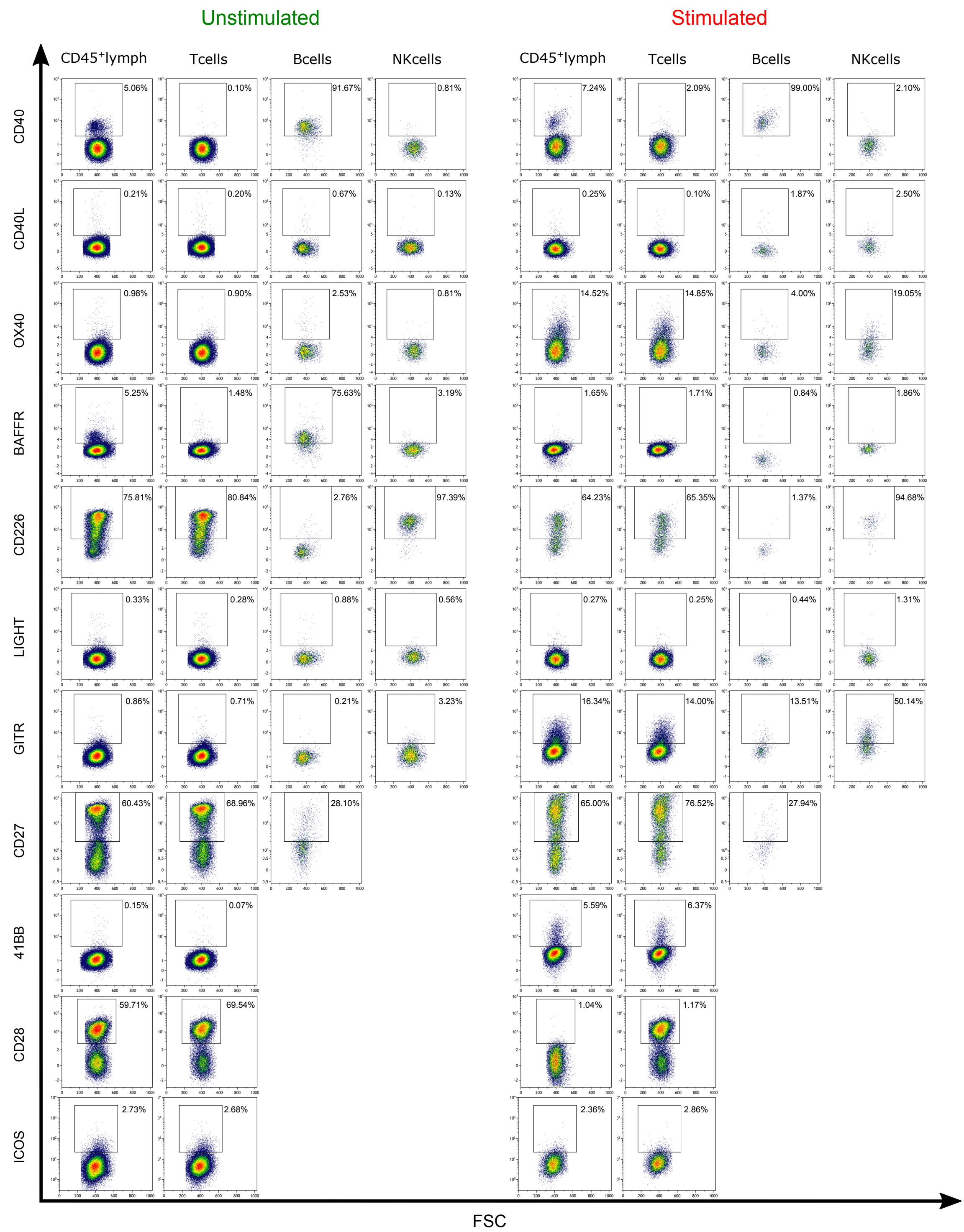

Supplementary Figure 15 Representative flow cytometry plots of co-stimulatory marker expression (CD40, CD40L, OX40, BAFFR, CD226, LIGHT, GITR, CD27, 41BB, CD28, ICOS) on unstimulated and stimulated healthy control PBMCs. Flow cytometry analyses of co-stimulatory molecule expression on the indicated immune subsets was performed before and after stimulation with anti-CD3 (Biolegend, USA, clone OKT3, 0.5 µg/ml) and anti-CD28 (Biolegend, USA, clone CD28.2, 2 µg/ml) for two days. The gating strategy is described in Supplementary Figure 12.

	HC PBMC				CA PBMC				TILs				References
	CD45⁺lymph	T cells	B cells	NK cells	CD45⁺lymph	T cells	B cells	NK cells	CD45⁺lymph	T cells	B cells	NK cells	
**PD-L2**	0.50% ± 0.38	0.43% ± 0.25	0.58% ± 0.47	0.18% ± 0.19	0.51% ± 0.35<br>p=0.8416	0.48% ± 0.33<br>p=0.6074	0.32% ± 0.23<br>p=0.0032	0.15% ± 0.21<br>p=0.2455	1.16% ± 0.67<br>p<0.0001	1.22% ± 0.76<br>p<0.0001	0.45% ± 0.45<br>p=0.1245	0.27% ± 0.39<br>p=0.6767	Zou W et al., Nat Rev Immunol., 2008 Jun;8(6):467-77<br>NCT02812875 (antagonistic)
**PD1**	21.14% ± 6.28	25.65% ± 7.97	1.09% ± 0.49	0.42% ± 0.57	19.73% ± 7.71<br>p=0.2988	25.25% ± 10.24<br>p=0.6672	1.44% ± 1.79<br>p=0.6711	0.89% ± 1.86<br>p=0.0244	51.12% ± 16.29<br>p<0.0001	64.02% ± 15.78<br>p<0.0001	2.96% ± 2.97<br>p<0.0001	3.49% ± 5.53<br>p<0.0001	Francisco L et al., Immunol Rev., 2010 Jul;236:219-242<br>Rizvi et al., Lancet Oncol, 2017 Jan;18(1):31-41
**PVR**	1.33% ± 0.39	0.94% ± 0.38	1.04% ± 0.45	0.98% ± 0.40	1.55% ± 1.10<br>p=0.9573	0.94% ± 0.90<br>p=0.1102	0.77% ± 0.37<br>p=0.0033	1.03% ± 0.96<br>p=0.2728	3.44% ± 4.39<br>p=0.0596	3.91% ± 5.85<br>p=0.0014	0.94% ± 1.69<br>p=0.0007	1.48% ± 2.37<br>p=0.5526	Kučan Brlić P et al., Cell Mol Immunol., 2019 Jan;16(1):40-52<br>NA
**TIGIT**	21.91% ± 5.52	20.62% ± 6.96	0.62% ± 0.44	46.07% ± 14.07	25.64% ± 8.70<br>p=0.0505	25.77% ± 8.73<br>p=0.0022	0.74% ± 2.28<br>p=0.0263	41.32% ± 16.12<br>p=0.2052	39.95% ± 16.15<br>p<0.0001	47.06% ± 18.18<br>p<0.0001	1.08% ± 1.01<br>p=0.0240	28.51% ± 17.58<br>p<0.0001	Harjunpää H et al., Clin Exp Immunol, 2020 May; 200(2):108-119<br>NCT02913313 (antagonistic)
**CD96**	31.10% ± 10.93	37.22% 13.78	2.92% ± 3.46	9.50% ± 4.82	28.15% ± 10.52<br>p=0.2050	34.81% ± 13.41<br>p=0.3889	3.48% ± 3.83<br>p=0.4891	12.69% ± 10.38<br>p=0.1258	33.54% ± 14.72<br>p=0.5397	39.94% ± 16.34<br>p=0.5088	2.80% ± 3.46<br>p=0.8558	36.90% ± 22.72<br>p<0.0001	Aguilera AR et al., Oncoimmunology, 2018 Feb;7(5):e1424677<br>NA
**BTLA**	48.10% ± 12.75	53.36% ± 12.84	99.34% ± 0.62	0.82% ± 0.62	48.16% ± 14.59<br>p=0.9778	51.55% ± 15.52<br>p=0.6583	94.63% ± 17.40<br>p=0.0121	1.72% ± 1.78<br>p<0.0001	13.43% ± 10.67<br>p<0.0001	11.30% ± 10.98<br>p<0.0001	43.55% ± 27.89<br>p<0.0001	2.75% ± 5.57<br>p=0.0397	Watanabe N et al., Nat Immunol, 2003 Jul;4(7):670-9<br>NCT04137900 (antagonistic)
**CD160**	0.19% ± 0.17	0.16% ± 0.17	0.38% ± 0.24	0.39% ± 0.39	0.24% ± 0.25<br>p=0.3572	0.20% ± 0.23<br>p=0.2679	0.35% ± 0.31<br>p=0.2773	0.47% ± 0.45<br>p=0.4874	0.47% ± 0.72<br>p=0.1750	0.36% ± 0.54<br>p=0.1871	1.06% ± 2.73<br>p=0.8485	0.73% ± 1.40<br>p=0.4916	Cai G et al., Nat Immunol, 2008 Feb;9(2):176-85<br>NA
**HVEM**	4.78% ± 3.43	3.30% ± 3.55	37.77% ± 18.80	0.61% ± 0.49	5.87% ± 3.57<br>p=0.1226	3.92% ± 3.52<br>p=0.2283	37.86% ± 17.55<br>p=0.9987	0.86% ± 0.91<br>p=0.2123	3.09% ± 3.87<br>p=0.0058	2.26% ± 3.74<br>p=0.1285	15.27% ± 13.12<br>p<0.0001	1.61% ± 5.98<br>p=0.9676	Inoue et al., Anticancer Res, 2015 Mar;35(3):1361-7<br>NA
**NKG2A**	6.93% ± 3.63	2.36% ± 1.49	0.35% ± 0.17	46.40% ± 19.77	8.11% ± 5.82<br>p=0.7351	2.77% ± 3.24<br>p=0.7252	1.09% ± 5.09<br>p=0.4956	48.26% ± 19.12<br>p=0.7992	11.50% ± 8.27<br>p=0.0094	7.39% ± 7.40<br>p<0.0001	3.33% ± 8.07<br>p=0.0022	11.66% ± 8.55<br>p<0.0001	André P et al., Cell, 2018 Dec;175(7):1731-1743<br>NCT02671435 (antagonistic)
**CD66ace**	0.66% ± 0.59	0.22% ± 0.16	2.61% ± 2.41	0.21% ± 0.22	1.27% ± 1.60<br>p=0.0131	0.86% ± 1.46<br>p<0.0001	3.63% ± 3.97<br>p=0.1686	0.43% ± 0.54<br>p=0.0274	12.38% ± 10.98<br>p<0.0001	13.71% ± 13.18<br>p<0.0001	9.46% ± 9.01<br>p<0.0001	3.53% ± 4.16<br>p<0.0001	Ortenberg R et al., Mol Cancer Ther, 2012 Jun;11(6):1300-10<br>NCT02346955 (antagonistic)
**Gal9**	0.09% ± 0.10	0.09% ± 0.09	0.23% ± 0.17	0.04% ± 0.06	0.10% ± 0.12<br>p=0.9300	0.10% ± 0.13<br>p=0.6134	0.21% ± 0.18<br>p=0.4160	0.06% ± 0.14<br>p=0.3996	0.48% ± 0.60<br>p<0.0001	0.38% ± 0.45<br>p<0.0001	1.01% ± 3.16<br>p=0.0704	1.79% ± 4.20<br>p<0.0001	Deshpande AM et al., Cancer Res, 2018 Apr 14-18<br>NA
**LAG3**	0.97% ± 1.03	0.90% ± 0.99	0.53% ± 0.87	0.66% ± 1.15	1.17% ± 1.30<br>p=0.5839	1.11% ± 1.40<br>p=0.7528	0.22% ± 0.16<br>p=0.0154	1.04% ± 1.39<br>p=0.0739	4.04% ± 4.83<br>p<0.0001	4.31% ± 5.16<br>p<0.0001	0.59% ± 0.60<br>p=0.0801	1.88% ± 3.79<br>p=0.0418	Anderson AC et al., Immunity, 2016 May;44(5):989-1004<br>NCT03250832 (antagonistic)
**Tim3**	1.64% ± 1.28	0.20% ± 0.14	1.46% ± 0.87	9.25% ± 8.38	2.53% ± 1.82<br>p=0.0184	0.34% ± 0.33<br>p=0.0774	1.50% ± 0.67<br>p=0.4647	13.74% ± 11.27<br>p=0.0689	5.53% ± 6.57<br>p=0.0004	5.38% ± 7.17<br>p<0.0001	0.81% ± 0.75<br>p=0.0005	8.55% ± 6.98<br>p=0.9826	Anderson AC et al., Immunity, 2016 May;44(5):989-1004<br>NCT02817633 (antagonistic)
**VISTA**	0.25% ± 0.10	0.04% ± 0.03	0.22% ± 0.16	0.05% ± 0.05	0.65% ± 1.02<br>p=0.0110	0.06% ± 0.07<br>p=0.0615	0.84% ± 6.30<br>p=0.8129	0.14% ± 0.42<br>p=0.0166	0.54% ± 1.51<br>p=0.3642	0.26% ± 1.67<br>p=0.0069	0.78% ± 1.07<br>p=0.0127	0.11% ± 0.31<br>p=0.1794	Gao J et al, Nat Med, 2017 May;23(5):551-555<br>NCT02812875 (antagonistic)
**CD244**	26.44% ± 8.49	17.61% ± 8.95	1.33% ± 0.87	98.55% ± 1.41	30.78% ± 13.59<br>p=0.1884	22.10% ± 13.80<br>p=0.2727	1.21% ± 0.99<br>p=0.3139	97.88% ± 2.41<br>p=0.1312	36.70% ± 17.24<br>p=0.0066	34.92% ± 15.94<br>p<0.0001	2.01% ± 4.40<br>p=0.4972	89.66% ± 10.71<br>p<0.0001	Agresta L et al., Front Immunol, 2018 Nov;9:2809<br>NA
**CD48**	71.35% ± 13.17	74.54% ± 12.82	86.36% ± 8.81	44.94% ± 20.61	81.47% ± 11.57<br>p=0.0010	85.48% ± 11.68<br>p=0.0004	90.89% ± 6.77<br>p=0.0176	57.58% ± 16.72<br>p=0.0037	83.13% ± 16.48<br>p=0.0001	85.49% ± 16.85<br>p=0.0002	90.23% ± 12.67<br>p=0.0035	63.45% ± 19.56<br>p=0.0001	Agresta L et al., Front Immunol, 2018 Nov;9:2809<br>NA
**CD305**	32.23% ± 14.80	26.61% ± 15.04	49.62% ± 16.79	65.00% ± 23.01	31.40% ± 14.72<br>p=0.8087	25.36% ± 13.29<br>p=0.9720	52.55% ± 18.61<br>p=0.4215	53.52% ± 25.48<br>p=0.0855	13.81% ± 10.11<br>p<0.0001	12.16% ± 9.16<br>p<0.0001	12.68% ± 14.99<br>p<0.0001	30.24% ± 18.82<br>p<0.0001	Meyaard L et al., Immunity, 1997 Aug;7(2):283-90<br>NA
**CD158a**	5.08% ± 6.21	2.38% ± 6.31		23.78% ± 14.67	3.76% ± 3.92<br>p=0.2615	0.76% ± 1.45<br>p=0.1829		18.37% ± 11.11<br>p=0.1143	3.90% ± 5.17<br>p=0.0956	1.17% ± 1.66<br>p=0.2324		12.28% ± 10.05<br>p=0.0002	Benson DM Jr et al., Blood, 2011 Dec;118(24):6387-91<br>NCT01222286 (antagonistic)
**CD158b**	9.41% ± 12.34	6.70% ± 14.25		33.01% ± 17.08	4.32% ± 5.00<br>p=0.0263	1.94% ± 4.25<br>p=0.2450		23.02% ± 13.37<br>p=0.0040	2.68% ± 3.24<br>p<0.0001	1.39% ± 1.73<br>p=0.9892		15.43% ± 9.67<br>p<0.0001	Benson DM Jr et al., Blood, 2011 Dec;118(24):6387-91<br>NCT01222286 (antagonistic)
**CD158k**	0.37% ± 0.25	0.36% ± 0.25		0.16% ± 0.17	0.33% ± 0.36<br>p=0.0887	0.30% ± 0.37<br>p=0.0591		0.47% ± 2.74<br>p=0.5353	0.55% ± 0.79<br>p=0.8375	0.35% ± 0.42<br>p=0.3456		0.61% ± 2.67<br>p=0.7674	Bagot M et al., Lancet Oncol, 2019 Aug;20(8):1160-1170<br>NCT03902184 (ADCC/ antagonistic)
**CTLA4**	1.04% ± 1.48	0.57% ± 0.92			2.14% ± 3.46<br>p=0.9137	1.35% ± 2.61<br>p=0.8723			1.77% ± 1.72<br>p=0.0012	1.74% ± 1.54<br>p<0.0001			Masteller EL et al., J Immunol, 2000 May;164(10):5319-27<br>NCT03515629 (antagonistic)
**CD39**	13.01% ± 5.95	6.01% ± 4.04			15.45% ± 7.72<br>p=0.2047	8.00% ± 5.50<br>p=0.0929			43.54% ± 19.85<br>p<0.0001	39.58% ± 22.25<br>p<0.0001			Allard B et al., Immunol Rev, 2017 Mar;276(1):121-144<br>NCT04306900 (antagonistic)
**CD73**	17.26% ± 7.04	17.02% ± 7.91			15.52% ± 7.54<br>p=0.2293	14.34% ± 7.46<br>p=0.1049			15.08% ± 9.10<br>p=0.1311	11.57% ± 7.54<br>p=0.0016			Allard B et al., Immunol Rev, 2017 Mar;276(1):121-144<br>NCT04148937 (antagonistic)
**CD40**	5.19% ± 2.70	0.18% ± 0.21	86.43% ± 8.35	0.47% ± 0.48	5.88% ± 4.12<br>p=0.8212	0.31% ± 0.20<br>p=0.0002	76.35% ± 16.32<br>p=0.0022	0.62% ± 0.53<br>p=0.1954	7.96% ± 7.31<br>p=0.2450	0.82% ± 0.96<br>p<0.0001	69.94% ± 20.49<br>p<0.0001	1.56% ± 3.33<br>p=0.2832	Bjorck P et al., J Immunother Cancer, 2015 Nov;3(Suppl 2):P198<br>NCT02482168 (agonistic)
**CD40L**	0.42% ± 0.27	0.41% ± 0.29	0.95% ± 0.44	0.21% ± 0.13	0.71% ± 0.67<br>p=0.0278	0.71% ± 0.64<br>p=0.0382	0.99% ± 0.48<br>p=0.5006	0.37% ± 1.20<br>p=0.0823	1.29% ± 1.40<br>p<0.0001	1.53% ± 1.83<br>p<0.0001	0.82% ± 0.75<br>p=0.0672	0.81% ± 2.55<br>p=0.9649	Pérez-Melgosa et al., J Immunol, 1999 Aug;163(3):1123-7<br>NA
**OX40**	2.02% ± 0.85	2.28% ± 1.03	2.42% ± 1.09	0.39% ± 0.30	3.55% ± 2.56<br>p=0.0053	4.39% ± 3.71<br>p=0.0044	2.19% ± 0.98<br>p=0.5380	0.59% ± 0.48<br>p=0.0098	8.06% ± 5.12<br>p<0.0001	9.86% ± 6.11<br>p<0.0001	1.40% ± 1.98<br>p<0.0001	1.35% ± 1.58<br>p<0.0001	Curti BD et al., Cancer Res, 2013 Dec;73(24):7189-7198<br>NCT02559024 (agonistic)
**BAFFR**	3.00% ± 1.64	0.51% ± 0.12	47.52% ± 22.56	0.33% ± 0.28	2.81% ± 2.71<br>p=0.1156	0.51% ± 0.19<br>p=0.8929	32.00% ± 25.85<br>p=0.0065	0.28% ± 0.30<br>p=0.1048	1.42% ± 2.31<br>p<0.0001	0.43% ± 0.20<br>p=0.0142	6.90% ± 10.56<br>p<0.0001	0.59% ± 1.26<br>p=0.0782	Guan Ng L et al., J Immunol, 2004 Jul;173(2):807-17<br>NA
**CD226**	78.75% ± 3.67	81.98% ± 4.35	11.65% ± 5.76	93.27% ± 7.42	74.77% ± 9.51<br>p=0.0961	79.37% ± 8.55<br>p=0.2028	11.03% ± 7.59<br>p=0.3372	92.29% ± 7.30<br>p=0.1882	50.71% ± 15.56<br>p<0.0001	58.94% ± 16.91<br>p<0.0001	4.75% ± 4.61<br>p<0.0001	55.65% ± 19.00<br>p<0.0001	Du X et al., Proc Natl Acad Sci USA, 2018 Dec;115(50):E11740<br>NCT04099277 (agonistic)
**LIGHT**	0.44% ± 0.35	0.34% ± 0.31	0.64% ± 0.59	0.37% ± 0.40	0.42% ± 0.35<br>p=0.6959	0.35% ± 0.32<br>p=0.5553	0.45% ± 0.45<br>p=0.0172	0.49% ± 1.38<br>p=0.8090	1.15% ± 1.42<br>p<0.0001	1.14% ± 1.42<br>p<0.0001	1.08% ± 4.56<br>p=0.1702	1.28% ± 1.65<br>p=0.0008	Ware C, Annu Rev Immunol, 2005;23:787-819<br>NA
**GITR**	2.79% ± 1.95	2.26% ± 1.63	0.60% ± 0.66	8.73% ± 6.37	3.59% ± 3.44<br>p=0.2881	2.83% ± 2.69<br>p=0.3479	0.43% ± 0.32<br>p=0.0292	9.66% ± 8.98<br>p=0.9345	12.45% ± 9.71<br>p<0.0001	13.55% ± 10.51<br>p<0.0001	1.15% ± 2.28<br>p=0.1686	20.05% ± 16.41<br>p=0.0021	Narumi K et al., Sci Rep, 2019 Apr;9(1):5562<br>NCT02583165 (agonistic)
**CD27**	64.49% ± 14.43	76.98% ± 16.30	35.48% ± 12.57		58.53% ± 15.11<br>p=0.0553	73.99% ± 16.17<br>p=0.4245	29.23% ± 16.01<br>p=0.0377		33.93% ± 17.02<br>p<0.0001	41.16% ± 20.14<br>p<0.0001	32.52% ± 18.97<br>p=0.3255		Hendriks J et al., J Exp Med, 2003 Nov;198(9):1369-80<br>NCT04081688 (agonistic)
**41BB**	0.60% ± 0.48	0.51% ± 0.38			0.92% ± 2.00<br>p=0.2133	0.85% ± 1.99<br>p=0.2162			5.91% ± 5.75<br>p<0.0001	6.61% ± 6.37<br>p<0.0001			Bartkowiak T et al., Front Oncol, 2015 Jun;5:117<br>NCT03258008 (agonistic)
**CD28**	65.79% ± 10.75	81.33% ± 12.64			59.63% ± 14.88<br>p=0.0915	78.75% ± 14.95<br>p=0.5749			48.28% ± 13.48<br>p<0.0001	64.18% ± 17.40<br>p<0.0001			Esensten JH et al., Immunity, 2016 May;44(5):973-88<br>NA
**ICOS**	3.35% ± 1.85	4.23% ± 2.43			3.29% ± 1.84<br>p=0.9448	4.41% ± 2.61<br>p=0.6922			17.86% ± 13.95<br>p<0.0001	23.39% ± 16.64<br>p<0.0001			Dong C et al., Nature, 2001 Jan;409(6816):97-101<br>NCT04128696 (agonistic)

Supplementary Table 1 Tumor-infiltrating lymphocytes show dysregulation of immune-checkpoint molecules. Mean percentages of immune-checkpoint molecule expression with standard deviation on CD45⁺, T cells, B cells and NK cells in healthy control PBMCs (HC PBMCs), cancer PBMCs (CA PBMCs) and tumor-infiltrating lymphocytes (TILs) are indicated. References for each molecule and a representative clinical study with antagonistic (red) or agonistic (green) agents underlying the classification of the molecules as co-inhibitory or co-stimulatory. Significant differences between HC PBMCs and CA PBMCs or TILs were calculated by unpaired, two-tailed Mann-Whitney test.

Supplementary Table 3 Patient characteristics

	Cases n=146		HNSCC n=29			NSCLC n=17			EGA n=16			CRC n=21		
**Age**														
Years	**67**		**66**			**71**			**77**			**66**		
Range	21-90		52-88			53-86			26-90			51-79		
**Sex**														
	**n**	**[%]**	**n**	**Total [%]**	**Type [%]**	**n**	**Total [%]**	**Type [%]**	**n**	**Total [%]**	**Type [%]**	**n**	**Total [%]**	**Type [%]**
Female	**78**	53%	**7**	5%	24%	**11**	8%	65%	**9**	6%	56%	**13**	9%	62%
Male	**68**	47%	**22**	15%	76%	**6**	4%	35%	**7**	5%	44%	**8**	5%	38%
**UICC Stage**														
I	**44**	30%	**1**	1%	3%	**5**	3%	29%	**1**	1%	6%	**8**	5%	38%
II	**34**	23%	**3**	2%	10%	**3**	2%	18%	**4**	3%	25%	**6**	4%	29%
III	**37**	25%	**8**	5%	28%	**8**	5%	47%	**7**	5%	44%	**5**	3%	24%
IV	**31**	21%	**17**	12%	59%	1	1%	6%	**4**	3%	25%	**2**	1%	10%
**Tumor**														
1	**40**	27%	**3**	2%	10%	**6**	4%	35%	**2**	1%	13%	**0**	0%	0%
2	**44**	30%	**9**	6%	31%	**5**	3%	29%	**1**	1%	6%	**8**	5%	38%
3	**46**	32%	**9**	6%	31%	**5**	3%	29%	**11**	8%	69%	**10**	7%	48%
4	**16**	11%	**8**	5%	28%	**1**	1%	6%	**2**	1%	13%	**3**	2%	14%
**Node**														
x	**25**	17%	**2**	1%	7%	**1**	1%	6%	**0**	0%	0%	**0**	0%	0%
0	**60**	41%	**10**	7%	34%	**7**	5%	41%	**4**	3%	25%	**15**	10%	71%
1	**31**	21%	**4**	3%	14%	**5**	3%	29%	**3**	2%	19%	**3**	2%	14%
2	**25**	17%	**11**	8%	38%	**4**	3%	24%	**6**	4%	38%	**3**	2%	14%
3	**5**	3%	**2**	1%	7%	**0**	0%	0%	**3**	2%	19%	**0**	0%	0%
**Metastasis**														
Yes	**11**	8%	**2**	1%	7%	**1**	1%	6%	**1**	1%	6%	**3**	2%	14%
No	**135**	92%	**27**	18%	93%	**16**	11%	94%	**15**	10%	94%	**18**	12%	86%
**Grading**														
N/A	**4**	3%	**0**	0%	0%	**0**	0%	0%	**0**	0%	0%	**0**	0%	0%
G1	**6**	4%	**0**	0%	0%	**0**	0%	0%	**0**	0%	0%	**0**	0%	0%
G2	**92**	63%	**20**	14%	69%	**10**	7%	59%	**9**	6%	56%	**17**	12%	81%
G3	**44**	30%	**9**	6%	31%	**7**	5%	41%	**7**	5%	44%	**4**	3%	19%

	RCC n=16			HCC n=13			OVCA n=7			TGCT n=4			UCC n=7			BCA n=16		
**Age**																		
Years	**65**			**74**			**73**			**48**			**75**			**52**		
Range	51-87			54-80			55-78			21-68			57-84			38-77		
**Sex**																		
	**n**	**Total [%]**	**Type [%]**	**n**	**Total [%]**	**Type [%]**	**n**	**Total [%]**	**Type [%]**	**n**	**Total [%]**	**Type [%]**	**n**	**Total [%]**	**Type [%]**	**n**	**Total [%]**	**Type [%]**
Female	**8**	5%	50%	**5**	3%	38%	**7**	5%	100%	**0**	0%	0%	**2**	1%	29%	**16**	11%	100%
Male	**8**	5%	50%	**8**	5%	62%	**0**	0%	0%	**4**	3%	100%	**5**	3%	71%	**0**	0%	0%
**UICC Stage**																		
I	**12**	8%	75%	**6**	4%	46%	**0**	0%	0%	**4**	3%	100%	**0**	0%	0%	**7**	5%	44%
II	**2**	1%	13%	**6**	4%	46%	**0**	0%	0%	**0**	0%	0%	**1**	1%	14%	**9**	6%	56%
III	**1**	1%	6%	**1**	1%	8%	**5**	3%	71%	**0**	0%	0%	**2**	1%	29%	**0**	0%	0%
IV	**1**	1%	6%	**0**	0%	0%	**2**	1%	29%	**0**	0%	0%	**4**	3%	57%	**0**	0%	0%
**Tumor**																		
1	**12**	8%	75%	**5**	3%	38%	**0**	0%	0%	**3**	2%	75%	**0**	0%	0%	**9**	6%	31%
2	**2**	1%	13%	**7**	5%	54%	**2**	1%	29%	**1**	1%	25%	**2**	1%	29%	**7**	5%	24%
3	**1**	1%	6%	**1**	1%	8%	**5**	3%	71%	**0**	0%	0%	**4**	3%	57%	**0**	0%	0%
4	**1**	1%	6%	**0**	0%	0%	**0**	0%	0%	**0**	0%	0%	**1**	1%	14%	**0**	0%	0%
**Node**																		
x	**15**	10%	94%	**2**	1%	15%	**0**	0%	0%	**4**	3%	100%	**0**	0%	0%	**1**	1%	6%
0	**0**	0%	0%	**10**	7%	77%	**1**	1%	14%	**0**	0%	0%	**3**	2%	43%	**10**	7%	63%
1	**1**	1%	6%	**1**	1%	8%	**6**	4%	86%	**0**	0%	0%	**3**	2%	43%	**5**	3%	31%
2	**0**	0%	0%	**0**	0%	0%	**0**	0%	0%	**0**	0%	0%	**1**	1%	14%	**0**	0%	0%
3	**0**	0%	0%	**0**	0%	0%	**0**	0%	0%	**0**	0%	0%	**0**	0%	0%	**0**	0%	0%
**Metastasis**																		
Yes	**2**	1%	13%	**0**	0%	0%	**2**	1%	29%	**0**	0%	0%	**0**	0%	0%	**0**	0%	0%
No	**14**	10%	88%	**13**	9%	100%	**5**	3%	71%	**4**	3%	100%	**7**	5%	100%	**16**	11%	100%
**Grading**																		
N/A	**0**	0%	0%	**0**	0%	0%	**0**	0%	0%	**4**	3%	100%	**0**	0%	0%	**0**	0%	0%
G1	**2**	1%	13%	**2**	1%	15%	**0**	0%	0%	**0**	0%	0%	**0**	0%	0%	**2**	1%	13%
G2	**12**	8%	75%	**10**	7%	77%	**0**	0%	0%	**0**	0%	0%	**1**	1%	14%	**13**	9%	81%
G3	**2**	1%	13%	**1**	1%	8%	**7**	5%	100%	**0**	0%	0%	**6**	4%	86%	**1**	1%	6%

Flow Cytometry Antibodies				
Fluorochrome	Target	Clone	Company	Catalogue
FITC	CD45	HI30	Biolegend	304038
PE	CD273 (PD-L2)	MIH18	Biolegend	345506
PE	CD226 (DNAM-1)	11A8	Biolegend	338306
PE	CD270 (HVEM)	122	Biolegend	318806
PE	CD258 (LIGHT)	T5-39	Biolegend	318706
PE	CD48	BJ40	Biolegend	336708
PE	CD197 (CCR7)	G043H7	Biolegend	353204
PE	CD152 (CTLA-4)	BNI3	BD Bioscience	555853
PE	CD69	FN50	Biolegend	310906
PE	CD86	IT2.2	Biolegend	305406
PE	CD158k	539304	R&D Systems	FAB2878P
PE-Dazzle	CD134 (OX40)	Ber-ACT35	Biolegend	350020
PE-Dazzle	CD155 (PVR)	SKII.4	Biolegend	337616
PE-Dazzle	CD154 (CD40L)	24-31	Biolegend	310840
PE-Dazzle	CD366 (Tim-3)	F38-2E2	Biolegend	345034
PE-Dazzle	CD244 (2B4)	C1.7	Biolegend	329522
PE-Dazzle	CD185 (CXCR5)	J252D4	Biolegend	356928
PE-Dazzle	CD25	M-A251	Biolegend	356126
PE-Dazzle	CD28	CD28.2	Biolegend	302942
PE-Dazzle	IgD	IA6-2	Biolegend	348240
PerCP-Cy5.5	CD40	5C3	Biolegend	334316
PerCP-Cy5.5	CD268 (BAFF-R)	11C1	Biolegend	316918
PerCP-Cy5.5	CD160	BY55	Biolegend	341210
PerCP-Cy5.5	Galectin-9	9M1-3	Biolegend	348910
PerCP-Cy5.5	CD305 (LAIR1)	NKTA255	Biolegend	342804
PerCP-Cy5.5	CD8	SK1	Biolegend	344710
PerCP-Cy5.5	CD127	A019D5	Biolegend	351322
PerCP-Cy5.5	CD24	ML5	Biolegend	311116
PerCP-Cy5.5	CD158b	DX27	Biolegend	312614
PE-Cy7	CD56	5.1H11	Biolegend	362510
PE-Cy7	CD45RA	HI100	Biolegend	304126
PE-Cy7	CD73	A2D	Biolegend	344010
PE-Cy7	CD27	O323	Biolegend	302838
Alexa Fluor 647	CD279 (PD1)	EH12.1	BD Bioscience	560838
Alexa Fluor 647	FoxP3	259D	Biolegend	320214
APC	CD96 (TACTILE)	REA195	Miltenyi	130-101-028
APC	CD159a (NKG2A)	REA110	Miltenyi	130-113-563
APC	CD66 ace	ASL-32	Biolegend	342308
APC	VISTA	730804	R&D Systems	FAB71261A
APC	CD21	Bu32	Biolegend	354906
APC	CD178	NOK-1	Miltenyi	130-096-458
Alexa Fluor 700	CD3	SK7	Biolegend	344822
Alexa Fluor 700	CD20	2H7	Biolegend	302322
APC-Fire 750	CD19	SJ25C1	Biolegend	363030
APC-Fire 750	CD4	SK3	Biolegend	344638
APC-Fire 750	CD8	SK1	Biolegend	344746
BV421	TIGIT	A15153G	Biolegend	372710
BV421	CD272 (BTLA)	MIH26	Biolegend	344512
BV421	CD223 (LAG3)	11C3C65	Biolegend	369314
BV421	CD357 (GITR)	108-17	Biolegend	371208
BV421	CD278 (ICOS)	C398.4A	Biolegend	313524
BV421	CD39	A1	Biolegend	328214
BV421	CD137 (4-1BB)	4B4-1	Biolegend	309820
BV421	CD38	HIT2	Biolegend	303526
BV421	CD158a	HP-3E4	BD Bioscience	564318
Zombie Aqua	fixable viability dye		Biolegend	423102
**Immunohistochemistry Antibodies**				
Target	Clone	Dilution/ Device/ Retrieval	Company	Catalogue
CD3	SP7	1:50/ BOND/ Citrate pH6	Thermo Fisher	RM-9107
CD8	C8/144B	1:200/ BOND/ Citrate pH6	Dako	M7103
MHC-I, HLA-A+B	EPR1394Y	1:300/ BOND/ Citrate pH6	Abcam	ab134189
CD274 (PD-L1)	28-8	1:100/ BOND/ EDTA pH8	Abcam	ab205921
Galectin-9	D9R4A	1:200/ BOND/ EDTA pH8	Cell Signaling	54330
CEACAM-1	D3R80	1:400/ BOND/ EDTA pH8	Cell Signaling	44464
CD155 (PVR)	D8A5G	1:200/ BOND/ EDTA pH8	Cell Signaling	81254
CD270 (HVEM)	2G6-2C7	1:200/ Ventana/ EDTA pH8	LS Bio	LS-B6186
VISTA	D1L2G	1:100/ BOND/ EDTA pH8	Cell Signaling	64953
HLA-E	MEM-E/02	1:200/ BOND/ Citrate pH6	Origene	SM3053P

Supplementary Table 4 Detailed antibodies and staining condition of flow cytometry and immunohistochemistry.

HUGO Gene	Alias	Accession	Position	Target Sequence	Isoforms	Isoforms Hit	Hit rate [%]	Design Remarks
B2M	Beta-2-Microglobulin	NM_004048.2	236-335	TACTGAAGAATGGAGAGAGAATTGAAAAAGTGGAGCATTCAGACTTGTCTTTCAGCAAGGACTGGTCTTTCTATCTCTTGTACTACACTGAATTCACCCC	3	3	100.00%	
CALR	Calreticulin	NM_004343.2	966-1065	TCTCCCGATCCCAGTATCTATGCCTATGATAACTTTGGCGTGCTGGGCCTGGACCTCTGGCAGGTCAAGTCTGGCACCATCTTTGACAACTTCCTCATCA	1	1	100.00%	
CANX	Calnexin	NM_001746.3	203-302	TGTTACTGGTGCTTGGAACTGCTATTGTTGAGGCTCATGATGGACATGATGATGATGTGATTGATATTGAGGATGACCTTGACGATGTCATTGAAGAGGT	14	14	100.00%	
ERAP1	Endoplasmic Reticulum Aminopeptidase 1	NM_001040458.1	755-854	CGAGCCCCTCCTTGTCGGGCTCCCGTACACAGTTGTCATTCACTATGCTGGCAATCTTTCGGAGACTTTCCACGGATTTTACAAAAGCACCTACAGAACC	15	15	100.00%	
ERAP2	Endoplasmic Reticulum Aminopeptidase 2	NM_001130140.1	156-255	AATTCATGTATTGAAAATATTGTTCAGACCCCATGTGACATAACTGGAGCCAGTGCAGTGCCATGAAGAACTACGAGATTAGCCTGGATATTAACTTGTC	7	7	100.00%	
FBXW7	F-Box And WD Repeat Domain Containing 7	NM_018315.4	1481-1580	TGGGTCATGTTGCAGCAGTCCGCTGTGTTCAATATGATGGCAGGAGGGTTGTTAGTGGAGCATATGATTTTATGGTAAAGGTGTGGGATCCAGAGACTGA	16	15	93.75%	sub-maximal isoform coverage due to non-overlapping transcripts
HLA-A	Major Histocompatibility Complex, Class I, A	XR_430999.1	1125-1224	AGCCTGGGCCCTGTGTGCCAGCACTTACTCTTTTGTAAAGCACCTGTTAAAATGAAGGACAGATTTATCACCTTGATTACAGCGGTGATGGGACCTGATC	5	1	20.00%	
HLA-B	Major Histocompatibility Complex, Class I, B	NM_005514.6	938-1037	CCCTGAGATGGGAGCCGTCTTCCCAGTCCACCGTCCCCATCGTGGGCATTGTTGCTGGCCTGGCTGTCCTAGCAGTTGTGGTCATCGGAGCTGTGGTCGC	1	1	100.00%	
HLA-C	Major Histocompatibility Complex, Class I, C	NM_002117.4	896-995	AGCTGGGAGCCATCTTCCCAGCCCACCATCCCCATCATGGGCATCGTTGCTGGCCTGGCTGTCCTGGTTGTCCTAGCTGTCCTTGGAGCTGTGGTCACCG	2	2	100.00%	
NLRC5	NLR Family CARD Domain Containing 5	NM_032206.4	3669-3768	TCCTGCTGGCCAACACCTTAAGCCTGTGTCCACGGGTTAAAAAGGTGGATCTCAGGTCCCTGCACCATGCAACTTTGCACTTCAGATCCAACGAGGAGGA	23	23	100.00%	
PDIA2	Protein Disulfide Isomerase Family A Member 2	NM_006849.2	1199-1298	CCTGATTGGGATCAGCGGCCAGTTAAGACCCTCGTGGGCAAGAATTTTGAGCAGGTGGCTTTTGACGAAACCAAGAATGTGTTTGTCAAGTTCTATGCCC	1	1	100.00%	
PDIA3	Protein Disulfide Isomerase Family A Member 3 (ERp57)	NM_005313.4	2329-2428	CTGTCCATGCCCTAAGGATGGGTTCCTGTTTATCCTTGCCACGCAGCTGAGCTTACTGCATGTTTATATCTCCCAAGGACTGTTCTCTGCTCAGAAATGC	1	1	100.00%	
PSMB1	Proteasome Subunit Beta 1	NM_002793.3	538-637	AGCTTTGATCCAGTAGGGTCTTACCAGAGAGACTCCTTCAAGGCTGGAGGCTCAGCAAGTGCCATGCTACAGCCCCTGCTTGACAACCAGGTTGGTTTTA	1	1	100.00%	
PSMB10	Proteasome Subunit Beta 10 (LMP10)	NM_002801.2	222-321	ACCATCGCGGGCCTGGTGTTCCAAGACGGGGTCATTCTGGGCGCCGATACGCGAGCCACTAACGATTCGGTCGTGGCGGACAAGAGCTGCGAGAAGATCC	1	1	100.00%	
PSMB2	Proteasome Subunit Beta 2	NM_002794.4	658-757	CTTCACACGCCGAAACCTGGCTGACTGTCTTCGGAGTCGGACCCCATATCATGTGAACCTCCTCCTGGCTGGCTATGATGAGCATGAAGGGCCAGCGCTG	3	3	100.00%	
PSMB5	Proteasome Subunit Beta 5	NM_001130725.1	427-526	CGTGGACAGTGAAGGGAACCGGATTTCAGGGGCCACCTTCTCTGTAGGTTCTGGCTCTGTGTATGCATATGGGGTCATGGATCGGGGCTATTCCTATGAC	4	3	75.00%	
PSMB8	Proteasome Subunit Beta 8 (LMP7)	NM_004159.4	1216-1315	ACTCACAGAGACAGCTATTCTGGAGGCGTTGTCAATATGTACCACATGAAGGAAGATGGTTGGGTGAAAGTAGAAAGTACAGATGTCAGTGACCTGCTGC	2	2	100.00%	
PSMB9	Proteasome Subunit Beta 9 (LMP2)	NM_002800.4	456-555	TCAGGTATATGGAACCCTGGGAGGAATGCTGACTCGACAGCCTTTTGCCATTGGTGGCTCCGGCAGCACCTTTATCTATGGTTATGTGGATGCAGCATAT	1	1	100.00%	
TAP1	Transporter 1, ATP Binding Cassette Subfamily B Member	NM_000593.5	2076-2175	GTGGCTGCAGTGGGACAAGAGCCACAGGTATTTGGAAGAAGTCTTCAAGAAAATATTGCCTATGGCCTGACCCAGAAGCCAACTATGGAGGAAATCACAG	2	2	100.00%	
TAP2	Transporter 2, ATP Binding Cassette Subfamily B Member	NM_000544.3	910-1009	GGCTTCCTTTAAATGCCAATGTGCTCTTGCGAAGCCTGGTGAAAGTGGTGGGGCTGTATGGCTTCATGCTCAGCATATCGCCTCGACTCACCCTCCTTTC	3	3	100.00%	
TAPBP	Tapasin	NM_003190.4	1083-1182	CATGGGGCCCATGGACCGGAAATGGGACCTTCTGGCTGCCTACAGTTCAACCCTTTCAGGAGGGCACCTATCTGGCCACCATACACCTGCCATACCTGCA	5	5	100.00%	
THOP1	Thimet Oligopeptidase 1	NM_003249.3	621-720	CTCCCCAGAGAGACTCAGGAAAACATCAAACGCATCAAGAAGAAGCTGAGCCTTCTGTGCATCGACTTCAACAAGAACCTGAACGAGGACACGACCTTCC	3	2	66.67%	
UBB	Ubiquitin B	NM_018955.2	411-510	GGAAGTGGAGCCCAGTGACACCATCGAAAATGTGAAGGCCAAGATCCAGGATAAAGAAGGCATCCCTCCCGACCAGCAGAGGCTCATCTTTGCAGGCAAG	6	6	100.00%	also targets UBBP4 [98%]
UBC	Ubiquitin C	NM_021009.4	314-413	GCGGAGGGATCTCCGTGGGGCGGTGAACGCCGATGATTATATAAGGACGCGCCGGGTGTGGCACAGCTAGTTCCGTCGCAGCCGGGATTTGGGTCGCAGT	1	1	100.00%	
CD160		NM_007053.3	723-822	AACAAAGACAACACCTTGAGTTCAGCCATAATGAAGGCACTCTCAGTTCAGGCTTCCTACAAGAAAAGGTCTGGGTAATGCTGGTCACCAGCCTTGTGGC	4	4	100.00%	
CD200		NM_005944.5	666-765	CCCTCGGTCAGGGATTGAAAATAGTACAGTGACTCTGTCTCACCCAAATGGGACCACGTCTGTTACCAGCATCCTCCATATCAAAGACCCTAAGAATCAG	19	19	100.00%	
CD274	Programmed Cell Death 1 Ligand 1 (PD-L1)	NM_014143.3	502-601	GCCCCATACAACAAAATCAACCAAAGAATTTTGGTTGTGGATCCAGTCACCTCTGAACATGAACTGACATGTCAGGCTGAGGGCTACCCCAAGGCCGAAG	4	4	100.00%	
CD47		NM_001777.3	898-997	GCCATATTGGTTATTCAGGTGATAGCCTATATCCTCGCTGTGGTTGGACTGAGTCTCTGTATTGCGGCGTGTATACCAATGCATGGCCCTCTTCTGATTT	14	13	92.86%	
CEACAM1	Carcinoembryonic Antigen Related Cell Adhesion Molecule 1 (CD66a)	NM_001712.4	509-608	TAAAGTCAGATCTTGTGAATGAAGAAGCAACTGGACAGTTCCATGTATACCCGGAGCTGCCCAAGCCCTCCATCTCCAGCAACAACTCCAACCCTGTGGA	7	7	100.00%	also targets CEACAM3/5/6 [>94%]
HLA-E	Major Histocompatibility Complex, Class I, E	NM_005516.4	1205-1304	TGTCTTAGGGGACTCTGGCTTCTCTTTTTGCAAGGGCCTCTGAATCTGTCTGTGTCCCTGTTAGCACAATGTGAGGAGGTAGAGAAACAGTCCACCTCTG	4	4	100.00%	
HLA-F	Major Histocompatibility Complex, Class I, F	NM_001098479.1	576-675	TGCGCTCCTGGACCGCGGCGGACACCGTGGCTCAGATCACCCAGCGCTTCTATGAGGCAGAGGAATATGCAGAGGAGTTCAGGACCTACCTGGAGGGCGA	13	13	100.00%	
HLA-G	Major Histocompatibility Complex, Class I, G	NM_002127.4	1181-1280	AAGAGCTCAGATTGAAAAGGAGGGAGCTACTCTCAGGCTGCAATGTGAAACAGCTGCCCTGTGTGGGACTGAGTGGCAAGTCCCTTTGTGACTTCAAGAA	5	3	60.00%	
IDO1	Indoleamine 2,3-Dioxygenase 1 (IDO)	NM_002164.5	1046-1145	AGTCAAATCCCTCAGTCCGTGAGTTTGTCCTTTCAAAAGGTGATGCTGGCCTGCGGGAAGCTTATGACGCCTGTGTGAAAGCTCTGGTCTCCCTGAGGAG	1	1	100.00%	
IL10	Interleukin 10	NM_000572.2	231-330	AAGGATCAGCTGGACAACTTGTTGTTAAAGGAGTCCTTGCTGGAGGACTTTAAGGGTTACCTGGGTTGCCAAGCCTTGTCTGAGATGATCCAGTTTTACC	1	1	100.00%	
LGALS9	Galectin 9	NM_002308.3	360-459	GCTGGGGGCCCGAGGAGAGGAAGACACACATGCCTTTCCAGAAGGGGATGCCCTTTGACCTCTGCTTCCTGGTGCAGAGCTCAGATTTCAAGGTGATGGT	9	9	100.00%	also targets LGALS9B and LGALS9C [>96%]
NECTIN1	PVRL1 (CD111)	NM_203285.1	221-320	GCTCTCGGCTTGACCGCATTCTTCCTCCCAGGCGTCCACTCCCAGGTGGTCCAGGTGAACGACTCCATGTATGGCTTCATCGGCACAGACGTGGTTCTGC	3	3	100.00%	
NECTIN2	PVRL2 (CD112)	NM_001042724.1	1121-1220	GCTTCGAGGAACCAGCCCTGATACCTGTGACCCTCTCTGTACGCTACCCTCCTGAAGTGTCCATCTCCGGCTATGATGACAACTGGTACCTCGGCCGTAC	2	2	100.00%	
PDCD1LG2	Programmed Cell Death 1 Ligand 2 (PD-L2)	NM_025239.3	643-742	AGGAAAATAAACACTCACATCCTAAAGGTTCCAGAAACAGATGAGGTAGAGCTCACCTGCCAGGCTACAGGTTATCCTCTGGCAGAAGTATCCTGGCCAA	2	2	100.00%	
PTEN	Phosphatase And Tensin Homolog	NM_000314.3	1676-1775	TGTGGTCTGCCAGCTAAAGGTGAAGATATATTCCTCCAATTCAGGACCCACACGACGGGAAGACAAGTTCATGTACTTTGAGTTCCCTCAGCCGTTACCT	3	3	100.00%	also targets PTENP1 [96%]
PVR	PVR Cell Adhesion Molecule (CD155)	NM_006505.3	605-704	GCTGCGGAATGCCTCGCTGAGGATGTTCGGGTTGCGCGTAGAGGATGAAGGCAACTACACCTGCCTGTTCGTCACGTTCCCGCAGGGCAGCAGGAGCGTG	4	4	100.00%	
SELPLG	Selectin P Ligand (PSGL1)	NM_001206609.1	327-426	CCTGCTTGCCCGGGACCGGAGACAGGCCACCGAATATGAGTACCTAGATTATGATTTCCTGCCAGAAACGGAGCCTCCAGAAATGCTGAGGAACAGCACT	2	2	100.00%	
SIGLEC9	Sialic Acid Binding Ig Like Lectin 9 (CD329)	NM_001198558.1	1053-1152	TGCAGAGCTCAGAACCCTCTCGGCTCTCAGCAGGTCTACCTGAACGTCTCCCTGCAGAGCAAAGCCACATCAGGAGTGACTCAGGGGGTGGTCGGGGGAG	9	9	100.00%	
TGFB1	Transforming Growth Factor Beta 1	NM_000660.3	1261-1360	TATATGTTCTTCAACACATCAGAGCTCCGAGAAGCGGTACCTGAACCCGTGTTGCTCTCCCGGGCAGAGCTGCGTCTGCTGAGGCTCAAGTTAAAAGTGG	2	2	100.00%	
TNFRSF14	TNF Receptor Superfamily Member 14 (HVEM/CD270)	NM_003820.3	660-759	CTGCAGCCCAGGCCACTTCTGCATCGTCCAGGACGGGGACCACTGCGCCGCGTGCCGCGCTTACGCCACCTCCAGCCCGGGCCAGAGGGTGCAGAAGGGA	10	10	100.00%	
VSIR	V-Set Immunoregulatory Receptor (VISTA)	NM_022153.1	186-285	ATCCCTGCTCTTCGCTCTCTTCCTGGCTGCGTCCCTAGGTCCGGTGGCAGCCTTCAAGGTCGCCACGCCGTATTCCCTGTATGTCTGTCCCGAGGGGCAG	1	1	100.00%	
ABCF1	ATP Binding Cassette Subfamily F Member 1	NM_001090.2	851-950	GATGTCCTCCCGCCAAGCCATGTTAGAAAATGCATCTGACATCAAGCTGGAGAAGTTCAGCATCTCCGCTCATGGCAAGGAGCTGTTCGTCAATGCAGAC	2	2	100.00%	
GUSB	Glucuronidase Beta	NM_000181.3	1900-1999	CCGATTTCATGACTGAACAGTCACCGACGAGAGTGCTGGGGAATAAAAAGGGGATCTTCACTCGGCAGAGACAACCAAAAAGTGCAGCGTTCCTTTTGCG	16	16	100.00%	
HPRT1	Hypoxanthine Phosphoribosyltransferase 1	NM_000194.1	241-340	TGTGATGAAGGAGATGGGAGGCCATCACATTGTAGCCCTCTGTGTGCTCAAGGGGGGCTATAAATTCTTTGCTGACCTGCTGGATTACATCAAAGCACTG	1	1	100.00%	
OAZ1	Ornithine Decarboxylase Antizyme 1	NM_004152.2	314-413	GGTGGGCGAGGGAATAGTCAGAGGGATCACAATCTTTCAGCTAACTTATTCTACTCCGATGATCGGCTGAATGTAACAGAGGAACTAACGTCCAACGACA	2	2	100.00%	
POLR2A	RNA Polymerase II Subunit A	NM_000937.2	3776-3875	TTCCAAGAAGCCAAAGACTCCTTCGCTTACTGTCTTCCTGTTGGGCCAGTCCGCTCGAGATGCTGAGAGAGCCAAGGATATTCTGTGCCGTCTGGAGCAT	1	1	100.00%	
SF3A1	Splicing Factor 3a Subunit 1	NM_001005409.1	236-335	CTTCTAAGCCAGTTGTGGGGATTATTTACCCTCCTCCAGAGGTCAGAAATATTGTTGACAAGACTGCCAGCTTTGTGGCCAGAAACGGGCCTGAATTTGA	1	1	100.00%	
SF3A3	Splicing Factor 3a Subunit 3	NM_006802.2	491-590	GAGGCACAAAACTTGGTGGAGTTCACAGATGAAGAGGGATATGGTCGTTATCTCGATCTCCATGACTGTTACCTCAAGTACATTAACCTGAAGGCATCTG	2	2	100.00%	
TBP	TATA-Box Binding Protein	NM_001172085.1	588-687	ACAGTGAATCTTGGTTGTAAACTTGACCTAAAGACCATTGCACTTCGTGCCCGAAACGCCGAATATAATCCCAAGCGGTTTGCTGCGGTAATCATGAGGA	2	2	100.00%	
ZNF143	Zinc Finger Protein 143	NM_003442.5	926-1025	TGAGCATGCAGGCTGTGGGAAGGCATTTGCAACAGGTTATGGATTAAAAAGTCACGTCAGAACTCATACAGGAGAAAAGCCATATCGGTGTTCGGAAGAT	12	12	100.00%	

Supplementary Table 5 Detailed code-set information with target sequence and hit rate of NanoString assays.

SUPPLEMENTARY METHODS

Isolation of PBMCs, TILs, NT and flow cytometry

Peripheral blood mononuclear cells (PBMCs) were isolated by Pancoll density-gradient centrifugation (PAN-Biotech, Germany). Tissue specimens were processed mechanically (gentleMACSDissociator, Miltenyi Biotech, Germany) and enzymatically (320 U/ml collagenase-IV, Worthington, USA and 100 U/ml DNAse-I, Applichem, Germany) to obtain a single-cell suspension containing the tumor-infiltrating lymphocytes (TILs). Cells were filtered using a 100 µm and a 70 µm cell-strainer and resuspended in fetal bovine serum (FBS) supplemented with 10% dimethyl sulfoxide (DMSO) and stored in liquid nitrogen until analysis. TILs and PBMCs were stained for 10-color flow cytometry (detailed antibody list, Supplementary Table 4). Intracellular FoxP3 staining was performed using the FOXP3 Fix/Perm buffer set according to the manufactures protocol (Biolegend, USA).

Automated immune-score analysis

High-resolution images of CD3 and CD8 stained slides were captured using a Leica SCN400 slide scanner (20X objective). Tumor front was delineated on digital scans by an experienced pathologist. The invasive margin (IM) was defined as an area of 350 µm ranging 50 µm from the tumor front to the center of the tumor (CT) and 300 µm to the healthy tissue. The center of the tumor (CT) comprised the whole tumor section excluding the invasive margin. IM and CT sections were separated and cropped into tiles of 1024x1024 pixels utilizing OpenSlide Python and the libvips image processing library. Staining artefacts and tissue folds were digitally excluded. Automated analysis of DAB-positive cells per area was performed using CellProfiler v 2.1.1 (CellProfiler Imaging Analysis, RRID:SCR_007358). A schematic overview of the immune-score pipeline is provided in Supplementary Figure 10. Counting accuracy was verified by reviewing all cropped tiles. Using the median $CD3^+$ or $CD8^+$ cell density of each cancer type as cut-off value, samples were classified as $CD3^{high}$ or $CD3^{low}$ and $CD8^{high}$ or $CD8^{low}$, respectively. Finally, patients were subdivided into five groups (IS0-ISIV) according to their $CD3^+$ and $CD8^+$ cell infiltration of IM and the CT as described [1]. Subgroups 0-I were termed “Immune-score low” and III-IV “Immune-score high”.

RNA isolation and NanoString

The tumor area was delineated on hematoxylin-eosin stained slides of formalin-fixed paraffin embedded (FFPE) sections containing tumor tissue and separate sections of corresponding healthy tissue, furthest from the tumor area by an experienced pathologist. RNA was isolated using the Maxwell® RSC RNA FFPE Kit (Promega, USA) according to the manufacturer’s instructions. RNA purity was measured by

Nano-Drop™ 2000c spectrophotometer (Thermo Fisher, USA). RNA was hybridized with a customized code-set of 54 genes on a T100 Thermal Cycler (Bio-Rad, USA) for 20h at 65°C (lid 70°C) and processed according to the manufacturer's protocol (detailed information on the customized code-set in Supplementary Table 5). RNA counts were normalized using 9 housekeeping genes and the included technical positive controls in each hybridization. Background signal was subtracted using the technical negative control of each hybridization reaction. Fold change was calculated using the RNA count of each tumor sample and the corresponding healthy tissue individually.

Flow cytometry controls

FACS-based detection of the included immune-regulatory molecules was validated using healthy control PBMCs. PBMCs were stained either unstimulated or after stimulation using 0.5 µg/ml anti-CD3 and 2 µg/ml anti-CD28 (both Biolegend, USA) for 2 days at 37°C (representative staining controls Supplementary Figure 13-15).

Statistical analyses and visualizations

Applicable statistical analyses were performed using GraphPad v8.3.0 (GraphPad Prism, RRID:SCR_002798, USA) as indicated in the corresponding figure legends. FACS plots were exported from Kaluza v2.1 (Kaluza, RRID:SCR_016182, Beckman Coulter, USA), graphs were generated using GraphPad v8.3.0 and figures were created using Inkscape v1.0beta1 (Inkscape, RRID:SCR_014479). Upset plots were generated using the UpsetR packages in R v3.6.3 (R Project for Statistical Computing, RRID:SCR_001905) and RStudio v 1.2.5033 (RStudio, RRID:SCR_000432). Hierarchical clustering and similarity matrices were generated using MORPHEUS (Morpheus by Broad Institute, RRID:SCR_017386).

References

1. Galon, J. *et al.* Towards the introduction of the 'Immunoscore' in the classification of malignant tumours. *J. Pathol.* **232**, 199–209 (2014).
