# Supplementary material for: Cancer-specific immune evasion and substantial heterogeneity within cancer types provide evidence for personalized immunotherapy
Source: NPJ Precis Oncol. 2021 Jun 16;5:52. doi: 10.1038/s41698-021-00196-x (PMC8208982; doi:10.1038/s41698-021-00196-x)
Supplement: Supplementary file 3 — Reporting Summary [file 41698_2021_196_MOESM3_ESM.pdf]

nature research

Corresponding author(s): Martin Thelen

Last updated by author(s): 14/5/21

Reporting Summary

Nature Research wishes to improve the reproducibility of the work that we publish. This form provides structure for consistency and transparency in reporting. For further information on Nature Research policies, see our Editorial Policies and the Editorial Policy Checklist.

Statistics

For all statistical analyses, confirm that the following items are present in the figure legend, table legend, main text, or Methods section.

n/a	Confirmed	
☐	☒	The exact sample size (*n*) for each experimental group/condition, given as a discrete number and unit of measurement
☐	☒	A statement on whether measurements were taken from distinct samples or whether the same sample was measured repeatedly
☐	☒	The statistical test(s) used AND whether they are one- or two-sided<br>*Only common tests should be described solely by name; describe more complex techniques in the Methods section.*
☒	☐	A description of all covariates tested
☐	☒	A description of any assumptions or corrections, such as tests of normality and adjustment for multiple comparisons
☐	☒	A full description of the statistical parameters including central tendency (e.g. means) or other basic estimates (e.g. regression coefficient) AND variation (e.g. standard deviation) or associated estimates of uncertainty (e.g. confidence intervals)
☐	☒	For null hypothesis testing, the test statistic (e.g. *F*, *t*, *r*) with confidence intervals, effect sizes, degrees of freedom and *P* value noted<br>*Give P values as exact values whenever suitable.*
☒	☐	For Bayesian analysis, information on the choice of priors and Markov chain Monte Carlo settings
☒	☐	For hierarchical and complex designs, identification of the appropriate level for tests and full reporting of outcomes
☒	☐	Estimates of effect sizes (e.g. Cohen's *d*, Pearson's *r*), indicating how they were calculated

Our web collection on statistics for biologists contains articles on many of the points above.

Software and code

Policy information about availability of computer code

Data collection	GalliosTM Kaluza (RRID:SCR_016700). Leica SCN400 Slidescanner for brightfield images, NanoString nCounter® Analysis System. For detailed decription of devices used for data aquisition, see "Methods" and Supplementary Methods"
Data analysis	Detailed software versions are described in "Methods" and "Supplementary Methods". Microsoft Excel (RRID:SCR_016137), Kaluza v2.1 (RRID:SCR_016182), Aperop ImageScope v12.4.0 (RRID:SCR_020993), Python v3.7 (RRID:SCR_008394), Matplotlib (RRID:SCR_008624), CellProfiler v2.1.1 (RRID:SCR_007358), GraphPad v8.3.0 (RRID:SCR_002798), Inkscape v1.0beta1 (RRID:SCR_014479), R v3.6.3 (RRID:SCR_001905), RStudio v1.2.5033 (RRID:SCR_000432), MORPHEUS (RRID:SCR_017386), nSolver Analysis Software (RRID:SCR_003420)

For manuscripts utilizing custom algorithms or software that are central to the research but not yet described in published literature, software must be made available to editors and reviewers. We strongly encourage code deposition in a community repository (e.g. GitHub). See the Nature Research guidelines for submitting code & software for further information.

Data

Policy information about availability of data

All manuscripts must include a data availability statement. This statement should provide the following information, where applicable:

- Accession codes, unique identifiers, or web links for publicly available datasets
- A list of figures that have associated raw data
- A description of any restrictions on data availability

The data that support the findings of this study are available from the corresponding author upon reasonable request.

Field-specific reporting

Please select the one below that is the best fit for your research. If you are not sure, read the appropriate sections before making your selection.

☒ Life sciences ☐ Behavioural & social sciences ☐ Ecological, evolutionary & environmental sciences

For a reference copy of the document with all sections, see nature.com/documents/nr-reporting-summary-flat.pdf

Life sciences study design

All studies must disclose on these points even when the disclosure is negative.

Sample size	146 treatment-naïve cancer patients were included. Samples from peripheral blood (n=137), tumor (n=141), corresponding healthy tissue (n=89) and formalin-fixed paraffin-embedded (FFPE) tumor (n=145) and paired corresponding heathy tissue (n=145) were collected.
Data exclusions	Samples containing less then hundred CD45 positive cells were excluded from flow cytometric subset analysis. Formalin-fixed paraffin-embedded (FFPE) blocks containing tumor with insufficient adjacent healthy tissue, were excluded from immune-score analysis.
Replication	*Describe the measures taken to verify the reproducibility of the experimental findings. If all attempts at replication were successful, confirm this OR if there are any findings that were not replicated or cannot be reproduced, note this and describe why.*
Randomization	*Describe how samples/organisms/participants were allocated into experimental groups. If allocation was not random, describe how covariates were controlled OR if this is not relevant to your study, explain why.*
Blinding	Flow cytometric analysis was performed in a blinded fashion, without knowledge of the sample source (peripheral blood mononuclear cells, tumor-infiltrating lymphocytes, or normal tissue-infiltrating lymphocytes) and the underlying cancer type.

Reporting for specific materials, systems and methods

We require information from authors about some types of materials, experimental systems and methods used in many studies. Here, indicate whether each material, system or method listed is relevant to your study. If you are not sure if a list item applies to your research, read the appropriate section before selecting a response.

Materials & experimental systems

n/a	Involved in the study	
☐	☒	Antibodies
☒	☐	Eukaryotic cell lines
☒	☐	Palaeontology and archaeology
☒	☐	Animals and other organisms
☐	☒	Human research participants
☐	☒	Clinical data
☒	☐	Dual use research of concern

Methods

n/a	Involved in the study	
☒	☐	ChIP-seq
☐	☒	Flow cytometry
☒	☐	MRI-based neuroimaging

Antibodies

Antibodies used	See Supplementary Table 4.
Validation	Healthy peripheral blood mononuclear cells (PBMCs) from three donors were stained before and after stimulation with 0.5 µg/ml anti-CD3 and 2 µg/ml anti-CD28 for 2 days to detect false negative staining. Results for a representative donor are shown in Supplementary Figures 13-15. Fluorescence-minus-one (FMO) experiments were performed for all included antibodies to detect false positive staining.

Human research participants

Policy information about studies involving human research participants

Population characteristics	For detailed patient characteristics, see Supplementary Table 3.
Recruitment	Only treatment naive cancer patients were included in this study.
Ethics oversight	Written informed consent was signed by all patients and this study was approved by our institutional ethics committee (no. 17-282)

Note that full information on the approval of the study protocol must also be provided in the manuscript.

Clinical data

Policy information about clinical studies

All manuscripts should comply with the ICMJE guidelines for publication of clinical research and a completed CONSORT checklist must be included with all submissions.

Clinical trial registration	*Provide the trial registration number from ClinicalTrials.gov or an equivalent agency.*
Study protocol	*Note where the full trial protocol can be accessed OR if not available, explain why.*
Data collection	*Describe the settings and locales of data collection, noting the time periods of recruitment and data collection.*
Outcomes	*Describe how you pre-defined primary and secondary outcome measures and how you assessed these measures.*

Flow Cytometry

Plots

Confirm that:

☒ The axis labels state the marker and fluorochrome used (e.g. CD4-FITC).

☒ The axis scales are clearly visible. Include numbers along axes only for bottom left plot of group (a 'group' is an analysis of identical markers).

☒ All plots are contour plots with outliers or pseudocolor plots.

☒ A numerical value for number of cells or percentage (with statistics) is provided.

Methodology

Sample preparation	Detailed sample preperation is decribed in "Methods" and "Supplementary Methods"
Instrument	Gallios Flow Cytometer (RRID:SCR_019639)
Software	Kaluza v2.1 (RRID:SCR_016182)
Cell population abundance	NA
Gating strategy	Detailed gating strategy is provided in Supplementary Figure 12

☒ Tick this box to confirm that a figure exemplifying the gating strategy is provided in the Supplementary Information.
